# Invasive *Streptococcus suis* isolated in Spain contain a highly promiscuous and dynamic resistome

**DOI:** 10.3389/fcimb.2023.1329632

**Published:** 2024-01-22

**Authors:** Cristina Uruén, Jorge Gimeno, Marina Sanz, Lorenzo Fraile, Clara M. Marín, Jesús Arenas

**Affiliations:** ^1^ Unit of Microbiology and Immunology, Faculty of Veterinary, University of Zaragoza, Zaragoza, Spain; ^2^ Institute Agrofood of Aragón-IA2, University of Zaragoza-CITA, Zaragoza, Spain; ^3^ Department of Animal Science, ETSEA, University of Lleida-Agrotecno, Lleida, Spain; ^4^ Department of Animal Production and Health, CITA, Zaragoza, Spain

**Keywords:** *Streptococcus suis*, antimicrobial resistance (AMR), antimicrobial resistant genes, mobile genetic elements, ICEs, IMEs, *Streptococcus* sp.

## Abstract

**Introduction:**

*Streptococcus suis* is a major pathogen for swine and human. Here we aimed to know the rates of antimicrobial resistance (AMR) in invasive *S. suis* isolates recovered along Spain between 2016 – 2021 and elucidate their genetic origin.

**Methods:**

Antibiotic susceptibility testing was performed for 116 isolates of different genetic backgrounds and geographic origins against 18 antibiotics of 9 families. The association between AMR and genotypes and the origin of the isolates were statistically analyzed using Pearson´s chi-square test and the likelihood ratio. The antimicrobial resistant genes were identified by whole genome sequencing analysis and PCR screenings.

**Results:**

High AMR rates (>80%) were detected for tetracyclines, spectinomycin, lincosamides, and marbofloxacin, medium (20-40%) for sulphonamides/trimethoprim, tiamulin, penicillin G, and enrofloxacin, and low (< 20%) for florfenicol, and four additional β-lactams. The occurrence of multidrug resistance was observed in 90% of isolates. For certain antibiotics (penicillin G, enrofloxacin, marbofloxacin, tilmicosin, and erythromycin), AMR was significantly associated with particular sequence types (STs), geographic regions, age of pigs, and time course. Whole genome sequencing comparisons and PCR screenings identified 23 AMR genes, of which 19 were previously reported in *S. suis* (*aph*(3’)-IIIa, *sat4, aadE, spw*, aac(6’)-Ie-*aph*(2’’)-Ia, *fexA, optrA, erm*(B), *mef*(A/E), *mrs*(D), *mph*(C), *lnu*(B), *lsa*(E), *vga*(F), *tet*(M), *tet*(O), *tet*(O/W/32/O), *tet*(W)), and 4 were novel (*aph*(2’’)-IIIa, *apmA, erm*(47), *tet*(T)). These AMR genes explained the AMR to spectinomycin, macrolides, lincosamides, tiamulin, and tetracyclines. Several genes were located on mobile genetic elements which showed a variable organization and composition. As AMR gene homologs were identified in many human and animal pathogens, the resistome of *S. suis* has a different phylogenetic origin. Moreover, AMR to penicillin G, fluoroquinolones, and trimethoprim related to mutations in genes coding for target enzymes (*pbp1a, pbp2b, pbp2x, mraY, gyrA*, *parC*, and *dhfr)*. Bioinformatic analysis estimated traits of recombination on target genes, also indicative of gene transfer events.

**Conclusions:**

Our work evidences that *S. suis* is a major contributor to AMR dissemination across veterinary and human pathogens. Therefore, control of AMR in *S. suis* should be considered from a One Health approach in regions with high pig production to properly tackle the issue of antimicrobial drug resistance.

## 1 Introduction

The Gram-positive coccus *Streptococcus suis* is a common inhabitant of the upper respiratory tract of pigs, but it is also the aetiological agent of streptococcal swine disease, a life-threatening infection characterized by the fast development of endocarditis, septicaemia, arthritis, and meningitis (Vötsch et al., 2018). It is a major cause of morbidity and mortality in sucking and transition pigs (Neila-Ibáñez et al., 2023), but can also cause infections in older pigs and sows. *S. suis* can be transmitted from pigs to humans by direct contact with animals, manipulation of pig meat, and consumption of raw meat. *S. suis* is also a zoonotic pathogen able to cause infections in humans as described in pigs (Goyette-Desjardins et al., 2014). Indeed, *S. suis* is one of the main meningitis-causing agents in several Asian countries.

Capsule is a major virulence factor for *S. suis*. The bacteria can express either one of the 29 different capsular polysaccharides (serotypes), but capsules 2, 3, 7, 8, 4, 1 1/2, and 9 are the most prevalent in clinical isolates from pigs (Goyette-Desjardins et al., 2014). Based on the antigenicity of the capsule, a serotyping scheme is used for routine diagnosis of strain virulence. Serotype distribution varies geographically, but serotype 2 predominates worldwide within clinical isolates (Goyette-Desjardins et al., 2014). In Spain, a recent study reported the most prevalent serotypes isolated from diseased pigs were 2 (21%), 1 (21%), 9 (19%), 3 (6%), and 7 (3%) (Petrocchi-Rilo et al., 2021). However, isolates of the same serotype show genetic heterogeneity, and thus genetic typing systems can better characterize this pathogen. Indeed, a Multi Locus Sequence Typing (MLST) scheme is available for *S. suis* and is globally used for epidemiological studies. Some sequence types (STs) are restricted to particular geographic regions (Goyette-Desjardins et al., 2014), for example, ST25 and ST28 in North America, while others are amply distributed, as ST1. Also, ST1 isolates were amply recovered from diseased people (Goyette-Desjardins et al., 2014).

Currently, the main immunization methods against *S. suis* in pig farming involve the use of commercial whole cells or bacterins prepared from clones recovered from diseased animals (Segura, 2015). However, the efficacy of bacterins is controversial (Corsaut et al., 2020), and there is a lack of tools to control safety, immunogenicity, or protective efficacy, leading to reliance on antibiotics as the primary defense against *S. suis* infections. This widespread antibiotic use contributes to high rates of antimicrobial resistance (AMR), particularly to tetracyclines, macrolides, β-lactams, aminoglycosides, sulfamethoxazole/trimethoprim (TMP), chloramphenicol, and fluoroquinolones, reported in clinical isolates recovered from diseased pigs and humans worldwide (Uruén et al., 2022). Many AMR genes were identified in *S. suis* (Dechêne-Tempier et al., 2021; Uruén et al., 2022). Some of them are placed on mobile genetic elements (MGE) such as integrative conjugative elements (ICEs), integrative mobile elements (IMEs), plasmids, transposons, genomic islands, or prophages. Thus AMR genes can easily migrate within strains (Dechêne-Tempier et al., 2021; Uruén et al., 2022). Notably, MGEs can be transferred from *S. suis* to different human streptococci pathogens such as *Streptococcus agalactiae* or *Streptococcus pyogenes* (Marini et al., 2015) or to pathogenic enterococci such as *Enterococcus faecalis* (Huang et al., 2016). This highlights *S. suis* as a relevant reservoir of AMR genes with potential implications for public health (Marini et al., 2015).

Spain is the highest pig producer in Europe, and it is hardly affected by *S. suis* infections. A recent study revealed that the percentage of units of sucking and nursery piglets clinically affected by *S. suis* is around 81% (Neila-Ibáñez et al., 2023). Antibiotics are highly used to treat and prevent *S. suis* infections in Spain (Neila-Ibáñez et al., 2023). Despite this microorganism is a zoonotic pathogen that can contribute to AMR dissemination, the rate of AMR for different antibiotics and their resistance mechanisms have been poorly studied. This knowledge is the basis for the design-built strategies to control *S. suis* disease in pigs and to minimize AMR spread. Therefore, we aimed here to know the rates of AMR for 18 different antibiotics in invasive isolates of *S. suis* recovered around Spain and to elucidate their genetic origin.

## 2 Material and methods

### 2.1 Bacterial isolates and growth conditions

A panel of 116 *S. suis* clinical isolates was selected in this study (**Supplementary Table S1**) from our *S. suis* collection described in our previous work (Uruén et al., 2024). The selective sampling criteria were *i*) only one isolate from the same farm, to avoid over-representation of endemic clones; *ii*) isolates should represent the most productive Autonomous Communities, *iii*) only isolates from internal organs or blood in which only one clone (serotype) was detected (to rule out the selection of opportunistic clones), *iv*) isolates recovered from the period 2014-2021 to have an indication of the current circulating *Ss*. 156 out of 843 isolates that matched the selective criteria were analyzed. All isolates were previously characterized in terms of serotyping, ST, and the presence of virulence-associated factors (Uruén et al., 2024).

Isolates were cultured onto BD™ Columbia Agar with 5% Sheep Blood (Columbia, Heidelberg, Germany) or Todd-Hewitt Agar (Oxoid Ltd., Hampshire, England) at 37°C in a candle jar for 18-24 hours. Todd-Hewitt liquid cultures were started from colonies grown on solid agar adjusted to an Optical Density of 600 nm (OD_600_) of 0.05. For antibiotic sensibility testing, bacteria were grown in Muëller-Hinton Broth (MHB) as described below.

### 2.2 Antibiotic susceptibility testing

Minimum Inhibitory Concentration (MIC) was determined in 18 antibiotics from 9 families using the broth microdilution method. Bacteria were thawed, propagated on blood agar, and incubated at 37°C in candle jars for 18-24 h. Three to five colonies were picked up and emulsified in Cation Adjusted MHB to obtain turbidity of 0.5 McFarland standard (Sensititre™ nephelometer V3011). Bacterial suspensions were further diluted in Cation Adjusted MHB with 2.5-5% Lysed Horse Blood to reach a final inoculum concentration of 5 x 10^5^ colony-forming units in 1 ml. A panel of 11 antibiotics (penicillin G, ampicillin, amoxicillin, ceftiofur, cefquinome, sulfamethoxazole/Timetropim (TMP), tiamulin, florfenicol, enrofloxacin, marbofloxacin, and doxycycline) was tested in customized 96-well microtiter plates (Sensititre, Trek Diagnostic Systems Inc., East Grinstead, UK). Plates were reconstituted by adding 100 μl/well of the inoculum. Besides, a panel of 7 antibiotics (spectinomycin, erythromycin, tilmicosin, tylosin, clindamycin, lincomycin, and tetracycline) were tested in 96-well microtiter U bottom plates (Deltalab, Barcelona, Spain). Antibiotic dilutions were prepared following the recommendations of ISO 20776-1:2019, and 100 μl of each antibiotic was added to each well together with the bacterial inoculum as depicted above. The MIC value was established as the lowest drug concentration inhibiting visible growth. *Streptococcus pneumoniae* (ATCC 49619™) was included as internal quality control following CLSI recommendations (2019). The resistance/susceptibility to each antibiotic was determined based on breakpoints established in EUCAST (EUCAST, 2023) and CLSI (CLSI, 2019), and when not available, ECOFF 95% or literature was used.

### 2.3 Statistical analysis

Associations between AMR, the genotype of isolates, geographical distribution, age of animals, and year of isolation were analyzed using Pearson’s chi-square test, however, when comparing two categories and more than 20% of the cells had a frequency lower than 5 units, the likelihood ratio test was used. Associations were considered statistically significant when the *p*-value was lower than 0.05. In addition, adjusted standardized residues (ASR) were calculated and analyzed. When the ASR value is higher than 1.96, the frequency is significantly higher than expected and the relationship is considered positively significant; if the ASR value is lower than -1.96, the frequency is significantly lower than expected, and the association is considered negatively significant. When the ASR value is between -1.96 and 1.96 the association between variables is not statistically significant. All statistical analyses were performed with SPSS software version 26 (IBM Corporation, Armonk, NY, USA). In some analyses the risk ratio (RR) was calculated as in (Viera, 2008). Shortly, RR was estimated with the formula $\frac{a÷\left(a+b\right)}{c÷\left(c+d\right)}$ where *a* is the number of resistant isolates with the mutations, *c* is the number of resistant isolates with no mutations, *b* is the number of susceptible isolates with the mutations, and *d* is the number of susceptible isolates with no mutations.

### 2.4 Chromosomal DNA extraction

The chromosomal DNA was obtained using the Chromosomal DNA extraction kit Wizard® Genomic DNA Purification Kit (Promega, USA), according to manufacturer instructions, and used as template DNA for PCRs and whole genome sequencing. The DNA integrity was evaluated in a 0.7% agarose gel, and the quality and quantity of DNA were measured with a Nanodrop spectrophotometer (Implen, Madrid, Spain) and a Qubit 4 fluorimeter (Thermo Fisher Scientific, DE, USA).

### 2.6 Whole genome sequencing and bioinformatics analyses

Whole genome sequencing was performed in 11 *S. suis* isolates (Ss_02, Ss_69, Ss_70, Ss_80_ Ss_92, Ss_93, Ss_100, Ss_121, Ss_156, Ss_166, and Ss_167). The selective criteria were *i*) isolates that showed resistance to most antibiotics tested in this study, particularly isolates with different AMR patterns, and *ii*) represent all major genetic clusters and some genetically unrelated singletons. This was also considering 19 previously sequenced genomes (Ss_08, Ss_20, Ss_21, Ss_22, Ss_24, Ss_45, Ss_46, Ss_48, Ss_51, Ss_52, Ss_53, Ss_72, Ss_84, Ss_106, Ss_107, Ss_109, Ss_115, Ss_124, and Ss_134) (Uruén et al., 2024). For whole genome sequencing, the Illumina HiSeq 2500 system was used. The preparation of the DNA-Seq libraries was performed at STAB Vida Lda (Caparica, Portugal), followed by sequencing by HiSeq 2500. Quality control of the raw reads was conducted using FastQC v.0.11.7 (https://www.bioinformatics.babraham.ac.uk/projects/fastqc/). Trimmomatic v.0.38 was employed for trimming, aiming to eliminate low-quality reads and adaptors (Bolger et al., 2014). The bowtie2 tool was used to remove any potential contamination by human DNA (*Homo sapiens* (b38):hg38). Lastly, the SPAdes tool was used to assemble the sequences, matching the trimmed short-read Illumina sequences. The quality of the assembly was assessed using Quast 5.0.2 (Gurevich et al., 2013). For pangenome relatedness, firstly, sequences of the 30 genomes of our collection and 8 genomes of reference strains were analyzed with Prokka tool (Seemann, 2014). The obtained data were used to construct a maximum-likelihood phylogenetic tree using the Roary tool to obtain accessory binary genes in Newick format (Page et al., 2015).

To identify the AMR genes from the 30 genomes of the isolates, the ABRicate tool against ResFinder and NCBI bacterial AMR gene database was used. ICEs and IMEs were identified with ICEberg 2.0 database. When required, DNA sequences were aligned with MAFFT (https://www.ebi.ac.uk/Tools/msa/mafft/). To search for traits of recombination, a full exploratory recombination scan using all methods of the program was carried out on DNA fragments with the RDP4 program (Martin et al., 2015). Recombination points were considered when 3 or more algorithm tests were positive. The non-synonymous/synonymous substitution rate ratio (dN/dS) was estimated for each gene individually. To do that, the gene nucleotide sequence was aligned with MAFFT, and a maximum-likelihood phylogeny tree was elaborated with MEGA11 (Tamura et al., 2021). The tree and coding sequences of each gene were introduced in HyPhy program (Kosakovsky Pond et al., 2020), and the single-likelihood ancestor counting method (SLAC) was carried out with a 95% confidence interval.

### 2.5 Detection of AMR genes by PCR

To identify AMR genes in the entire population, PCR screenings were performed targeting those AMR genes detected in our genomes (described above) and those reported in the literature as highly prevalent in different studies. Together, a panel of 38 AMR genes (including antibiotic targeted genes) were examined, comprising a*ph*(3’)-IIIa*, ant1, aacA, aadE, ant*(9’)-Ia*, aac*(6’)-Ie-*aph*(2’’), *sat4, spw, apmA, aph*(2’’)-IIIa*, pbp2x, pbp2b, pbp1a, mraY, fexA, optrA, gyrA, parC, erm*(B), *mef*(A/E), *mrs*(D), *erm*(47), *mph*(B), *mph*(C), *cfr, lnu*(B), *lsa*(E), *vga*(F), *dhfr, tet*(O), *tet(*M), *tet*(T), *tet*(L), *tet*(K), *tet*(W), *tet*(40), *tet*(44), *tet*(O/W/32/O). For PCR assays, 1 µl of chromosomic DNA, 0.25 µM of different primer combinations (**Supplementary Table S2**), and 400 µM dNTPs, were mixed with 0.5 U Taq DNA polymerase and PCR buffer (Biotools, Madrid, Spain). The mix was first incubated for 10 min at 94°C, followed by 30 cycles of 45 sec at 94°C, 30 sec at the annealing temperature described in **Supplementary Table S2**, 1 min per 1 kb of expected PCR product size at 72°C (**Supplementary Table S2**), and a final extension of 7 min at 72°C. The reactions were performed in a thermocycler (Biometra TRIO, Madrid, Spain). Amplicons were visualized in 1% agarose gels stained with Green®Nucleic Acid Stain (Sigma-Aldrich, Darmstadt, Germany) in an iBright Imaging System (Thermo Fisher Scientific, DE, USA). All PCR products obtained in this work were purified for sequencing using PCR Product Pre-Sequencing Kit (Thermo Fisher Scientific, DE, USA), and, when required, sequenced at STAB Vida Lda (Caparica, Portugal). The sequence of *pbp2x, pbp2b, pbp1a, mraY, dhfr, gyrA*, and *gyrB* was extracted from the whole genome sequencing analysis and analyzed independently. Partial sequencing was performed in *pbps* and *mraY* genes in isolate Ss_104, in *dhfr* gene in isolates Ss_82, Ss_85, Ss_104, and Ss_138, in *gyrA* and *parC* genes in isolates Ss_09, Ss_168, and Ss_170. When required, DNA sequences were translated with MEGA and used for sequence comparisons.

## 3 Results

### 3.1 Origin of the isolates

A total of 116 invasive isolates of *S. suis* from diseased pigs with symptoms of streptococcal swine disease were studied here (**Supplementary Table S1**). Isolates were recovered from pigs located in farms of 13 Spanish Autonomous Communities, comprising high swine producers (8-3x10^6^ pigs/year): Aragón (*n*=43), Cataluña (*n*=24), Castilla y Leon (*n*=16); moderate swine producers (1–3x10^6^ pigs/year): Andalucía (*n*=5), Castilla La Mancha (*n*=8), Murcia (*n*=8), Galicia (*n*=2), Valencia (*n*=4); and, low swine producers (< 1x10^6^ pigs/year): Cantabria (*n*=1), Extremadura (*n*=1), Madrid (*n*=1), Navarra (*n*=2), País Vasco (*n*=1). The sampling period was from 2016 to 2021. Isolates were from suckling piglets (*n*=11), transition pigs (*n*=74), and fattening pigs (*n*=3), but the age of the host was non-defined for 28 isolates. All isolates were previously characterized by ST, serotype, and virulence factor profile (**Supplementary Table S1**) as published in our previous work (Uruén et al., 2024). The four most frequent STs were ST1 (*n*=28), ST123 (*n*=22), ST29 (*n*=10), and ST3 (*n*=6). Also, few isolates were of ST1552 (*n*=3), ST24 (*n*=2), ST1642 (*n*=2), and ST94 (*n*= 3), and 40 belonged each one to a single ST, including ST13, ST16, ST17, ST198, ST141, ST198, ST949, ST1621, ST1622, ST1623, ST1624, ST1625, ST1626, ST1627, ST1628, ST1629, ST1630, ST1631, ST1632, ST1633, ST1634, ST1635, ST1636, ST1637, ST1638, ST1639, ST1640, ST1641, ST1644, ST1645, ST1649, ST1650, ST1651, ST1652, ST1653, ST1654, ST1822, ST1824, ST1825, ST2275. These STs were grouped in six phylogenetic eBurst Groups (eBGs): eBG1 (*n*=42), eBG2 (*n*=3), eBG3 (*n*=4), eBG4 (*n*=12), eBG5 (*n*=33), otherwise singletons (*n*=22) [characterized in Uruén et al. (2024)]. eBG2, eBG3, and eBG6 isolates belong to clonal complex (CC) CC16, CC24, and CC1628/1633, respectively. All isolates of eBG1 belong to CC1, but one isolate is unrelated. All isolates of eBG4 belong to CC29 but one is of CC28. eBG5 contain isolates of CC94 (n=4), CC123 (n=28), and one unrelated. Moreover, isolates were distributed in 11 serotypes, where serotype 9 prevailed (*n*=33), followed by serotype 1 (*n*=21) and serotype 2 (*n*=13). 14 isolates were non-tippable. The core genome of the 30 isolates used in this study and 8 public genomes was analyzed phylogenetically (**Supplementary Figure S1**). Two main clusters were generated. A cluster was constituted by all isolates of eBG1 and public genomes A7, O1/7, S10 and 2016UMN29656, all of them of CC11 but of different STs (ST1, ST3 and ST1642) and serotypes (1, 2, 1/2, 9 and 14). The other cluster was sub-grouped into two clusters. One cluster was constituted of two isolates and the public genome of 861160. They were of eBG2 (CC16) and singletons (CC20) that belonged to different serotypes (2, 4, 9) and STs (ST29, ST16, ST949). The remaining cluster contained singletons, and isolates of eBG5, eBG4, and eBG5. Isolates of eBG5 were of CC123 and CC94 and of serotypes 9 and 4 and eBG4 (CC29, ST29, non-tippable) and clustered together with genome SH1510. These genomes were phylogenetically related to isolates of eBG3 (CC24, ST1637, and ST24) than singletons. Hence, the collection of isolates studied here is very diverse regarding isolation origin (geographic location, host, and isolation year) and genetic background.

### 3.2 AMR profiles

**Figure 1:**
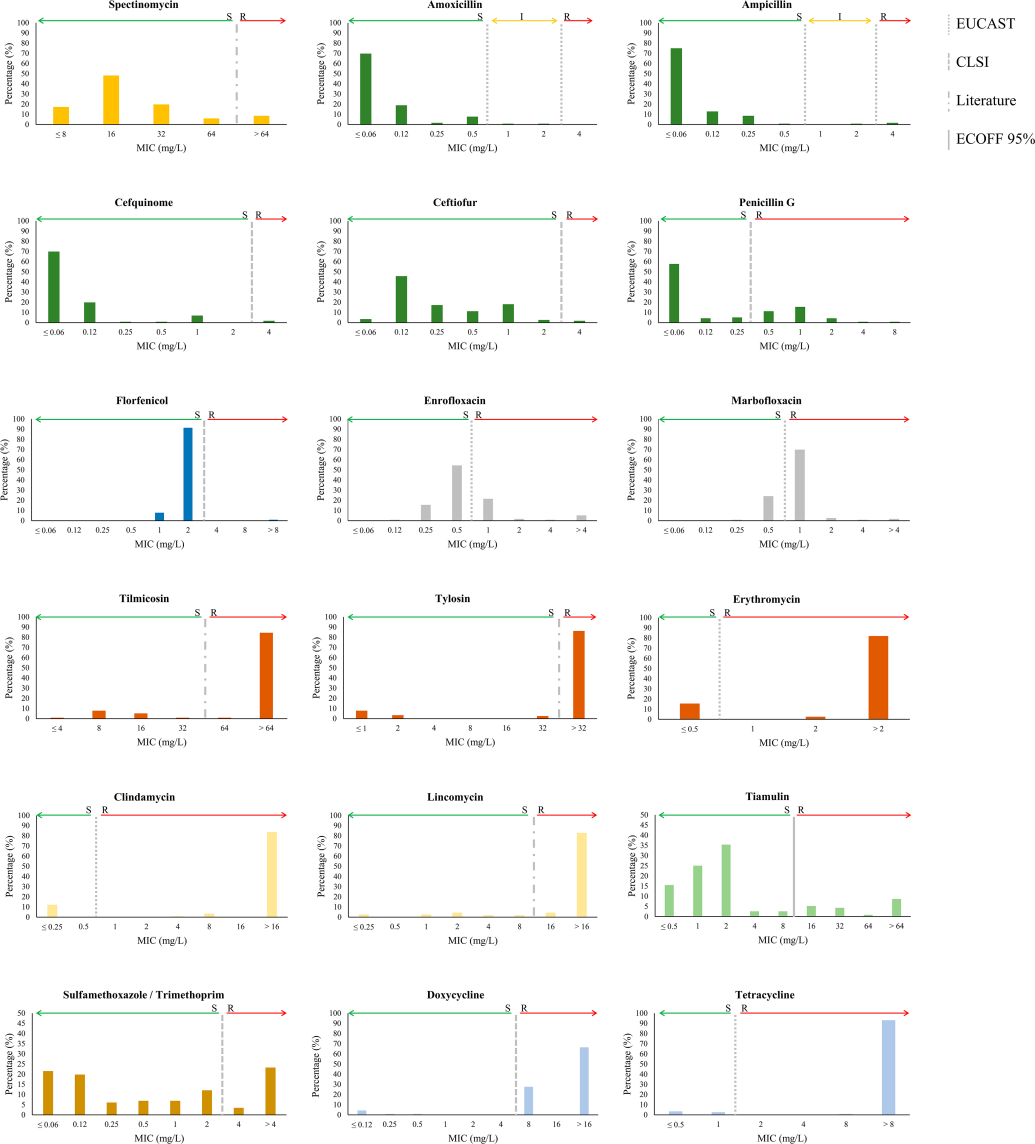
Distribution of MIC values for 18 antibiotics tested in 116 invasive *S. suis* isolates. Antibiotics of the same class are similarly coloured; the aminoglycosides (yellow), the β-lactams (dark green), the amphenicols (dark blue), the fluoroquinolones (grey), the lincosamides (light yellow), the macrolides (brown), the pleuromutilins (light green), the sulphonamides/TMP (ochre), and the tetracyclines (light blue). Breakpoints of resistance are indicated with vertical lines as determined by EUCAST, CLSI, literature reports, and ECOFF 95%.

The susceptibility of *S. suis* isolates against 18 antibiotics of 9 families was measured by the MIC. Included were one aminoglycoside (spectinomycin), five β-lactams (amoxicillin, ampicillin, cefquinome, ceftiofur, and penicillin G), one amphenicol (florfenicol), two fluoroquinolones (enrofloxacin, marbofloxacin), two lincosamides (clindamycin and lincomycin), one pleuromutilin (tiamulin), three macrolides (tilmicosin, tylosin, and erythromycin), sulfamethoxazole/TMP, and two tetracyclines (doxycycline and tetracycline). Amoxicillin, cefquinome, ceftiofur, and penicillin G are widely used to treat *S. suis* infections, the remaining antibiotics are of extensive use in the pig production industry to treat a variety of bacterial infectious diseases. The MIC value and the resulting phenotype for each isolate are listed in **Supplementary Table S1**, and the distribution of the MICs within the population is summarized in **Figure 1**. Results evidence variations in the distribution of the population for the different antibiotics. Hence, the distribution of MIC values for macrolides, tetracyclines, florfenicol, sulfamethoxazole/TMP, and clindamycin was bimodal, suggesting clear differences between susceptible and resistant populations. In contrast, the MIC distribution for fluoroquinolones and spectinomycin was normal, and for β-lactams, tiamulin, and lincosamides was right-skewed. Considering established and/or calculated breakpoints, high AMR rates were detected for tetracyclines (93%), macrolides (84%, 85%, 86% for erythromycin, tilmicosin, and tylosin, respectively), lincosamides (88% and 97% for clindamycin and lincomycin, respectively), and the fluoroquinolone marbofloxacin (81%). Medium AMR rates were detected for sulfamethoxazole/TMP (29%), tiamulin (19%), β-lactam penicillin G (32%), and enrofloxacin (30%). For florfenicol (5%), and the remaining β-lactams (2%) (amoxicillin, ampicillin, cefquinome, and ceftiofur), AMR rates were low. The occurrence of multidrug resistance, *i.e*., simultaneous AMR to antibiotics of at least three different families, was identified in 90% of the population. Up to 32 multi-resistance patterns were detected (**Supplementary Figure S2**). The most prevalent pattern (27% of isolates) was multi-resistance to fluoroquinolones, lincosamides, macrolides, and tetracyclines, although some isolates of this group were susceptible to certain antibiotics of the cited families. Conversely, isolate Ss_166, which belonged to this group, exhibited resistance to 14 antibiotics. The remaining patterns contained around or less than 10% of the isolates.

### 3.3 Distribution of AMR

The association between AMR, MLST-based genotypes, and the origin of the isolates was statistically analyzed. The high AMR rates for tetracyclines, macrolides, and lincosamides prevented significant associations with these antibiotics. But statistically significant associations were found with other antibiotics; for instance, the AMR rate for penicillin G was significantly high within isolates of ST123 (91%; *p*<0.001, ASR=6.7), all of them recovered from very distant geographic locations (**Supplementary Table S1**). ST123 belongs to the phylogenetic group eBG5 and is constituted of several other STs (*i.e*., ST94, ST1621, ST1622, ST1632, ST1640, ST1641, ST1650 and ST1652), 60% of which were susceptible to penicillin G. Thus, apparently, AMR for penicillin G is higher in ST123 isolates than the rest isolates. In contrast, AMR for penicillin G was significantly low in ST1 and ST29 isolates, both of them of eBG1. Also, AMR rates for enrofloxacin (48.1%; *p*=0.001, ASR=3.3), and marbofloxacin (100%; *p*=0.001, ASR=3.4) were significantly high in ST1 isolates. Finally, the AMR rates for clindamycin and tiamulin were also high in isolates of eBG5 (97%; *p*=0.035, ASR=1.9), eBG6 (100%; *p*<0.001, ASR=2.9), and singletons (65%; *p*<0.001, ASR=5.8). Notably, the AMR rates for tilmicosin and erythromycin varied within geographic regions; it was significantly high in isolates recovered from Aragón (93%; ASR=1.8), Cataluña (91%; ASR=1), Castilla La Mancha (87%; ASR=0.2), and Murcia (87%; ASR=0.3). Also, high AMR rates for erythromycin occurred in isolates recovered from Aragón (93%; ASR=1.9) and Cataluña (91%; ASR=1.1). Yet, certain Autonomous Communities exhibited high AMR rates for some antibiotics, but these associations had low statistical power, including AMR rates for spectinomycin in the Valencian Community (50%; *p*>0.05, ASR=2.8) or for penicillin G in Aragón (44.2%; *p*>0.05; ASR=2). Interestingly, the AMR rate for tiamulin was significantly high in fattening (66.7%; *p*=0.032; ASR=2.1) and suckling pigs (45.5%; *p*=0.032; ASR=2.4) as compared to transition pigs. When the AMR rates were compared within isolation years, the resistance to most β-lactams (amoxicillin, ampicillin, ceftiofur, and cefquinome) increased significantly over time (*p*=0.015 - 0.027; ASR=3.7). Together, our statistical analysis shows that the AMR rates for certain antibiotics varied according to genetic background (penicillin, enrofloxacin, marbofloxacin, clindamycin, and tiamulin), place of isolation (tilmicosin, erythromycin), growth stage of the host (tiamulin), and isolation year (penicillin G) of the isolates.

### 3.4 Identification of AMR determinants

To identify AMR genes in our population, we analyzed the genome of a subset of 30 *S. suis* isolates using bioinformatics tools. We make use of the genome of 19 isolates that were recently sequenced by us. Additionally, we sequenced 11 novel isolates (see details in material and methods). Isolates were resistant to multiple antibiotics, including aminoglycosides (*n*=4), β-lactams (*n*=10), fluoroquinolones (*n*=16), lincosamides (*n*=28), macrolides (*n*=25), pleuromutilins (*n*=9), sulphonamides/TMP (*n*=8), tetracyclines (*n*=29) (**Supplementary Figure S1**). A total of 19 AMR genes were identified, comprising *aph*(3’)-IIIa, *sat4, aadE, spw, aph*(2’’)-IIIa*, apmA, fexA, optrA, erm*(B)*, erm*(47), *mef*(A/E)*, lnu(*B)*, lsa*(E), *tet*(M)*, tet*(O), *tet*(W), *tet*(O/W/32/O), *tet*(T), and *vga*(F). All those genes were previously reported in drug-resistant *S. suis* (Dechêne-Tempier et al., 2021; Uruén et al., 2022), except for *apmA, aph*(2’’)-IIIa, *erm*(47), *tet*(T)) that were detected in other species (**Table 1**). Then, specific multi-PCR assays targeting the aforementioned genes were performed in the entire population. In addition, we analyzed 12 genes reported as prevalent in AMR *S. suis*, comprising *ant1, aacA, ant*(9’)-Ia, *aac*(6’)-Ie-*aph*(2’’), *mrs*(D), *mph*(C), *cfr, tet*(L), *tet*(K), *tet*(40), *tet*(44).

**Table 1:** AMR genes detected in 116 invasive isolates of *S. suis* recovered in Spain and their association with antibiotic resistance.

AMR genes	Reported in *S. suis*	Found in other bacteria (NCBI)^1,2^	Antibiotic family	Antibiotic tested	+/R	+/S	*p* value*^3^
*aph*(3**’**)-IIIa	Liang et al., 2021; Yongkiettrakul et al., 2021.	ER, EFaeca, EFaeci, StaA, CJ	Aminoglycosides	Spectinomycin	4/10	10/106	0.026
*sat4*	Liang et al., 2021; Yongkiettrakul et al., 2021.	EFaeca, EFaeci, StaA, EH, StrPar, Csp, CD, StrPy, CC	Aminoglycosides	Spectinomycin	1/10	8/106	–
*aadE a.k.a.* (*ant*(6)-Ia)	Hadjirin et al., 2021. Marini et al., 2015; Liang et al., 2021.	EH, ER, LM, StaA, EFaeci, EFaeca, StrA, EG, StrPar, CD, StrD	Aminoglycosides	Spectinomycin	9/10	29/106	0.026
*spw*	Chen et al., 2021.	EFaeca, EFaeci, ER, LM, StaA, StrA, StrMu, StrD, CD	Aminoglycosides	Spectinomycin	9/10	3/106	<0.001
*aph*(2**’’**)-IIIa	This study	EG, EFaecalis, CJ, ECas, CC	Aminoglycosides	Spectinomycin	2/10	0/106	0.002
*apmA*	This study	StaA, EFaeca	Aminoglycosides	Spectinomycin	1/10	0/106	0.029
*fexA*	Petrocchi-Rilo et al., 2021	EFaeca, EBac, StaS, EFaeci, ECol, StaA, BCer, StaC	Amphenicols	Florfenicol	0/1	1/115	–
*optrA*	Yongkiettrakul et al., 2021;	EFaeca, StaS, EFaeci, EH, StaA,	Amphenicols	Florfenicol	1/1	1/115	0.003
			Lincosamides	Clindamycin	2/102	0/14	–
				Lincomycin	2/102	0/14	–
*erm*(B)	Huang et al., 2016; Huang et al., 2016; Chen et al., 2021	EFaeci, EFaeca, CC, EH, EG,StrPn, StrPy, StrO, StrMi, StrMu	Macrolides	Erythromycin	98/99	0/17	<0.001
				Tilmicosin	98/99	0/17	<0.001
				Tylosin	98/100	0/16	<0.001
			Lincosamides	Clindamycin	97/102	1/14	<0.001
				Lincomycin	97/102	1/14	<0.001
*erm*(47)	This study	HK	Macrolides	Erythromycin	0/99	1/17	–
				Tilmicosin	1/99	0/17	–
				Tylosin	0/100	1/16	–
*mef*(A/E)	Chen et al., 2013	StrPy, StrPn, StrA, StrMi, BCel, BU, EFaeci	Macrolides	Erythromycin	2/99	0/17	–
				Tilmicosin	2/99	0/17	–
				Tylosin	2/100	0/16	–
*lnu*(B)	Bojarska et al., 2016	StaA, EFaeca, EH, EFaeci, EG, StrMu, Llno	Lincosamides	Clindamycin	5/102	0/14	–
				Lincomycin	5/102	0/14	–
*lsa*(E)	Bojarska et al., 2016	EFaeca, EFaeci, ER, LM, StaA, StrA, EG, EH, StrMu, Llno	Lincosamides	Clindamycin	5/102	0/14	–
				Lincomycin	5/102	0/14	–
			Pleuromutilines	Tiamulin	5/25	0/91	<0.001
*vga*(F)	Hadjirin et al., 2021	StrPar	Lincosamides	Clindamycin	21/102	0/14	0.014
				Lincomycin	21/102	0/14	0.014
			Pleuromutilines	Tiamulin	21/25	0/91	<0.001
*tet*(M)	Chen et al., 2013.	StrPar, CD, EFaeca, EFaeci, StrPn, EH, StrO, StaA, StrCr	Tetracyclines	Doxycycline	7/109	0/7	–
				Tetracycline	7/109	0/7	–
*tet*(O)	Chen et al., 2013; Huang et al., 2016	StrA, StrD, StrPn, APP, StrCa, Esp, StrPar, Efaeca, StrPy, CC, CJ, StrMu, Csp, SEn	Tetracyclines	Doxycycline	98/109	0/7	<0.001
				Tetracycline	98/109	0/7	<0.001
*tet*(O/W/32/O)	Chen et al., 2013	StrPas, EFaeca, StrG, EBac	Tetracyclines	Doxycycline	3/109	0/7	–
				Tetracycline	3/109	0/7	–
*tet*(W)	Chen et al., 2013	APy, LJ, CD, LInt, Corsp, CC	Tetracyclines	Doxycycline	3/109	0/7	–
				Tetracycline	3/109	0/7	–
*tet*(T)	This study	Csp, StaA, EFaeca, StrPy, HK, StrCa	Tetracyclines	Doxycycline	1/109	0/7	–
				Tetracycline	1/109	0/7	–

The population was distributed in resistant- (R) or susceptible (S) isolates for each antibiotic. The number of isolates with particular AMR gene (“+”) of each population is indicated.

^1^EFaeca, *Enterococcus faecalis*; EFaeci, *Enterococcus faecium*; StaA, *Staphylococcus aureus*; StrA, *Streptococcus agalactiae*; CC, *Campilobacter coli*; CJ, *Campilobacter jejuni*; StrPn, *Streptococcus penumoniae*; StrG, *Streptococcus gallolyticus*; CD, *Clostridioides difficile*; LIno, *Listeria inocua*; LJ, *Lactobacillus johnsonii*; ER, *Erysipelothrix rhusiopathiae*; EG, *Enterococcus gallinarum*; ECas, *Enterococcus casseliflavus*; LS, *Lactobacillus salivarius*; ECol, *Escherichia coli*; EBac, *Enterococcaceae bacterium*; StaS, *Staphylococcus sciuri*; BCer, *Bacillus cereus*; StaC, *Staphylococcus cohnii*; EH, *Enterococcus hirae*; StreP, *Streptococcus pasteurianus*; HK, *Helcococcus kunzii*; Psp, *Peptoniphilus* sp.; StrPy, *Streptococcus pyogenes*; StrMi, *Streptococcus mitis*; BCel, *Bacteroides cellulosilyticus*; BU, *Bacteroides uniformis*; LM, *Listeria monocytogenes*; StrPar, *Streptococcus parasuis*; SE, *Salmonella enterica*; StrCr, *Streptococcus cristatus*; StrMu, *Streptococcus mutans*; StrO, *Streptococcus oralis*; StrD, *Streptococcus dysgalactiae*; Csp, *Clostridium sp.*; APP, *Actinobacillus pleuropneumoniae*; StrCa, *Streptococcus canis*; Esp, *Enterococcus* sp.; StrPas, *Streptococcus pasteurianus*; APy, *Arcanobacterium pyogenes*; Lint, *Lawsonia intracellularis*; Corsp, *Corynebactiruim* sp.; SE, *Salmonella enterica*. ^2^Bacteria with more than 98% coverage and 92.07% of identity in BLAST analyses are listed. ^3^Statistical significant associations between the presence of AMR genes and resistant/susceptible profiles by indicating the p-value.

#### 3.4.1 Aminoglycosides

Aminoglycoside resistance in *S. suis* is produced by enzymatic modification of aminoglycosides, target site modification, and efflux pumps. Aminoglycoside modifying enzymes include Streptothricin N-acetyltransferase (*sat4*), diverse aminoglycoside O-nucleotidyltransferases (*ant, spw*), aminoglycoside O-phosphotransferases (*aph*) or aminoglycoside N-acetyltransferase–O-phosphotransferases (*ant*-*aph*). *aph*(3’)-IIIa, *sat4*, *spw*, *aph*(2’’)-IIIa, *apmA*, and *aadE* genes were detected in our population, and their distribution is indicated in **Table 1**. Interestingly, the *spw* gene was present in 9 out of 10 (90%) streptomycin-resistant isolates as compared to 3 out of 106 (3%) streptomycin-susceptible isolates. Sequencing of the *spw* gene in three spectinomycin-susceptible and three spectinomycin-resistant isolates showed 100% amino acid identity among isolates. Also, the promoter region had no mutations, except for a spectinomycin-susceptible isolate (Ss_125) that had few nucleotide alterations (**Supplementary Figure S3A**). These mutations may cause a lack or reduced gene expression for this isolate but did not clarify the lack of phenotype for the remaining two susceptible isolates. The *aph*(3´)-IIIa gene was present in 36% of the spectinomycin-resistant isolates and 9% of spectinomycin-susceptible isolates. In contrast, *aadE* gene was present in 90% of spectinomycin-resistant isolates and 28% of spectinomycin-susceptible isolates. The *sat4* gene was present in 9% and 7% of susceptible and resistant isolates, respectively. *apmA* and *aph*(2´)-IIIa were uniquely detected in one and two spectinomycin-resistant isolates, respectively. Statistical analysis revealed that *aph*(3´)-IIIa, *aadE, spw*, *aph*(2”)-IIIa and *apmA* were significantly associated with spectinomycin-resistant isolates (*p*<0.05, **Table 1**). Notably, *spw* showed the strongest association (*p*<0.001). Together, these data indicate that spectinomycin resistance is mainly caused by *spw, aadE*, and *aph*(3´)-IIIa. Interestingly, high MIC values were correlated with the co-existence of more than two AMR genes (*p*<0.001), indicative that their accumulation increases spectinomycin resistance.

**Figure 2:**
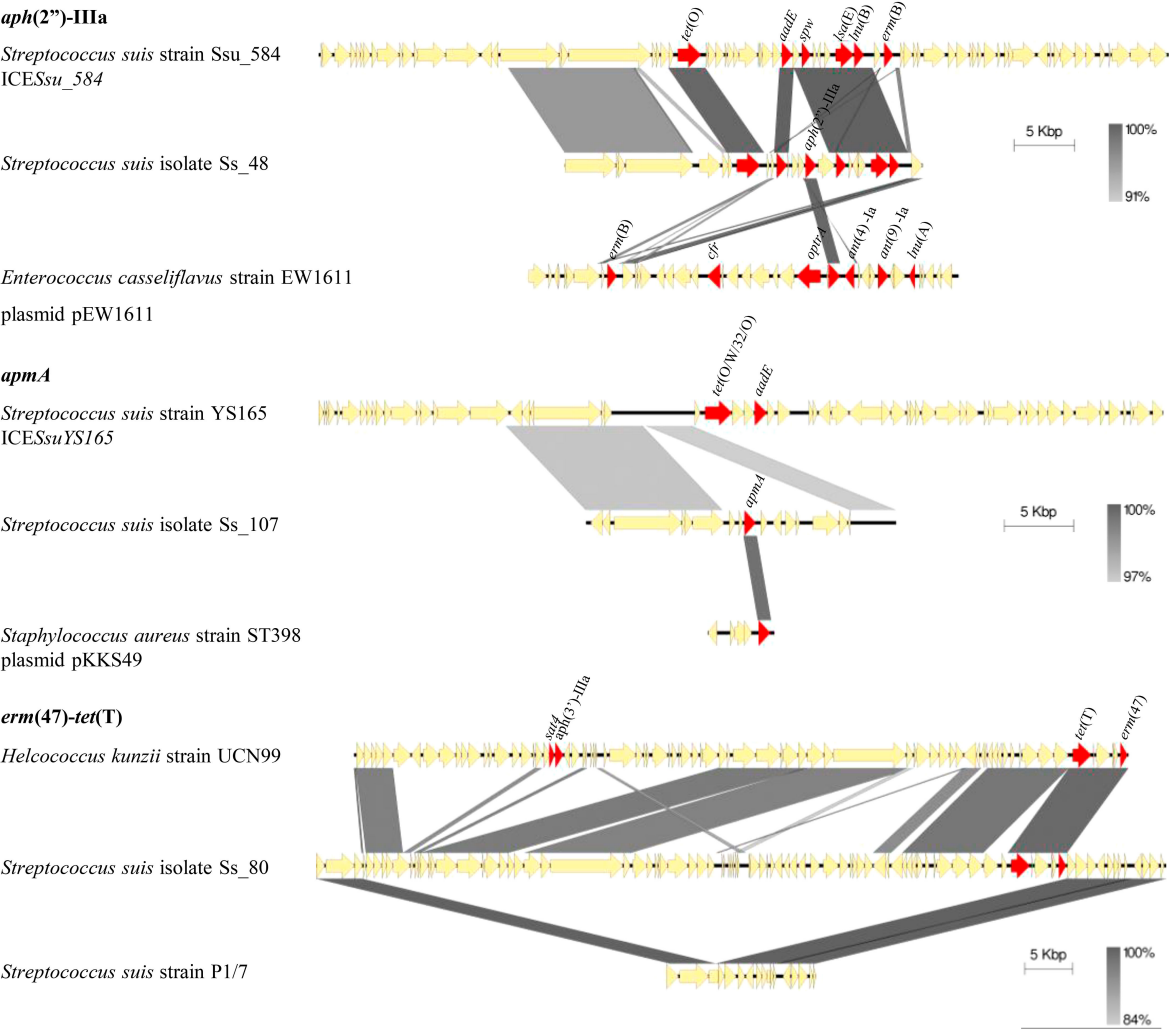
Genetic organization of *aph*(2’’)-IIIa, *ampA*, and *erm*(47) genes in *S. suis* isolates and other public genomes as indicated. AMR genes are red coloured and gene names are indicated.

The *aph*(2”)-IIIa and *apmA* genes were not reported in *S. suis* yet. To explore their origin, both genes, and their genetic context, were examined in isolates Ss_48 and Ss_107 that carry *aph*(2´)-IIIa and *apmA*, respectively, and compared with public genomes (**Figure 2**). The plasmid pEW1611 of *Enterococcus casseliflavus* strain EW1611 contains *aph*(2”)-IIIa, but their flanking regions have very little homology with those in *S. suis* isolate Ss_48. In contrast, the genome of *S. suis* strain Ssu_584 which lacked *aph*(2”)-IIIa, contained 22,324 bp with 97% of homology with several flanking regions of *aph*(2´)-IIIa in Ss_48 (**Figure 2**). This region corresponded to an ICE that carried multiple AMR genes including *tet*(O), *aadE*, *spw*, *lnu*(B), *lsa*(E), *erm*(B). Besides that, the gene *apmA* is located in the plasmid pKKKS49 of *Staphylococcus aureus* strain ST398, but their flanking regions lack any homology to the genome sequences of isolate Ss_107. Interestingly, the genome of *S. suis* YS165 contains an ICE (ICE*SsuYS165*) that lacks *apmA* but harbors a sequence of 13,091 bp with 97% of nucleotide homology with several flanking regions of *apmA* of isolate Ss_107 (**Figure 2**). Together, these analyses suggest that *aph*(2´)-IIIa and *apmA* were transferred from another bacteria to *S. suis* through MGEs.

#### 3.4.2 β-lactams

**Figure 3:**
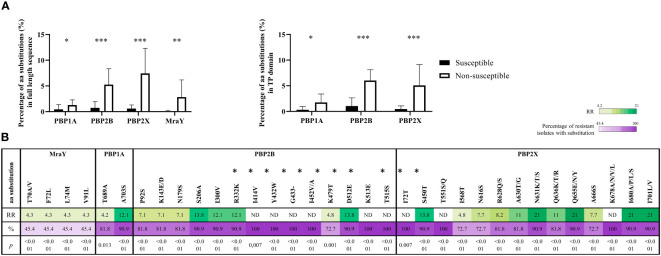
Genetic differences of Penicillin G resistant and susceptible isolates. **(A)** Amino acidic variability of penicillin-binding proteins (PBPs) and MraY proteins in resistant and susceptible *S. suis* isolates. The variability is shown for the full-length proteins (left graft) and transpeptidase domain (right graft). **(B)** Risk ratio (RR) for amino acid alterations in the sequences of MraY, PBP1A, PBP2B, and PBP2X. A total of 32 alterations were significantly associated and were present in 70% of penicillin G-resistant isolates (*p*< 0.05). Values non-defined (ND) correspond to alterations present in all resistant isolates, and therefore RR could not be calculated. The colour scale is proportional to the RR value. Mutations located in transpeptidase domain are indicated with an asterisk.

In Gram-positive bacteria, the resistance to β-lactams is mostly caused by mutations in the sequences that code for transpeptidase domains of Penicillin Binding Proteins (PBPs), which are targets for β-lactams, and *mraY* that code for an enzyme involved in the synthesis of a peptidoglycan precursor. There are up to six *pbp* genes (*pbp1a, pbp1b, pbp2a, pbp2b, pbp2x, pbp3*) in *S. suis*, but mutations in *pbp1a, pbp2b, pbp2x* and *mraY* were mainly related to β-lactams resistance (Bamphensin et al., 2021; Hadjirin et al., 2021). Hence, to investigate the origin of penicillin G resistance in our isolates, the sequence of *pbps* and *mraY* were analyzed in 31 *S. suis* isolates, including 20 penicillin G-susceptible and 11 penicillin G-resistant isolates. Compared with the *S. suis* genome of P1/7, which is susceptible to β-lactams, a total of 399 mutation sites were identified in the 31 isolates analyzed, comprising 37, 128, 185, and 49 sites in PBP1A, PBP2B, PBP2X, and MraY, respectively. Importantly, a significant abundance of amino acid substitutions occurred in PBP1A, PBP2B, PBP2X, and MraY (1.3%, 5.3%, 7.4% and 2.8%, respectively) of penicillin G-resistant isolates compared to penicillin G-susceptible isolates (0.4%, 0.7%, 0.6% y 0.1%, respectively) (*p*<0.05) (**Figure 3A**); many of these mutations occurred in the transpeptidase domain (**Figure 3A**). Notably, 301 substitutions were exclusively present in penicillin G-resistant isolates (9 in PBP1A, 90 in PBP2B, 157 in PBP2X, and 45 in MraY), and the most prevalent were G727D (36.4%) in PBP1A; A562D/P (63.6%) and Q668E/L, Q678T/P/N, Q685N/H, and H688Y (54.5%) in PBP2B; N631K/T/S, Q655E/N/Y, I680A/P/L/S, and I701L/V (90.9%) in PBP2X; A630T/G and Q636K/T/R (81.8%), N616S and A666S (72.7%), and T70A/V, F72L, L74M, and V91L (45.5%) in MraY. A total of 30 and 4 mutations in PBPs and MraY, respectively, were present in at least 70% and 40% of penicillin G-resistant isolates, respectively. Indeed, these mutations were statistically associated with penicillin G resistance (*p*<0.05). Then, the RR was calculated. An RR value higher than 1 is interpreted as an increased probability of the association between the genotypic event and the phenotype, and an RR lower than 1 as a negative association. A high RR was detected in 1, 4 and 5 mutations in PBP1A, PBP2B, and PBP2X, respectively (**Figure 3B**). Notably, no RR could be calculated for 6 mutations in PBP2B and 3 in PBP2X due to their high prevalence in resistant isolates and low in susceptible isolates (indicated as ND in **Figure 3B**). Most of these substitutions are located in the sequences that encode for the transpeptidase domain of the cited enzymes (**Figure 3B**).

Because of the large abundance of mutations found in PBPs, the level of genetic conservation of *pbps* and *mraY* genes was analyzed by calculating the dN/dS substitutions. dN/dS is a metric parameter commonly interpreted in terms of the amount of amino acid diversity generated by codon substitutions (Del Amparo et al., 2021). It is widely used to evaluate selection in protein coding sequences in a large variety of microorganisms, including viruses (Meyer et al., 2015) and bacteria (Rocha, 2006; Arenas et al., 2008). Positive selection was considered when the dN/dS is higher than 1, and negative selection when dN/dS is lower than 1. Positively or negatively coding sites were considered when the significance level was less than 0.05. The dN/dS of *pbp1a*, *pbp2x*, *pbp2b*, and *mraY* ranged from 0.13 to 0.05 (**Table 2**) evidencing negative selection for amino acid replacement. Actually, several negative selection sites were identified in all genes. Yet, *pbp2x* contained a positive selection site (Q636K/T/R) with a high prevalence in resistant isolates (**Figure 3B**). Then, to understand the origin of the mutations, the presence of putative recombination events was analyzed. Many putative recombination breakpoints (**Supplementary Table S3**) were estimated and located within the *pbp1a*, *pbp2b*, and *pbp2x-mraY* and their flanking regions (**Supplementary Figure S4**). Together, our analysis supports that penicillin G resistance in our isolates could be caused by polymutations in *pbp* and *mraY* genes, some of which could be likely acquired by horizontal genetic transfer.

**Table 2:** Estimated dN/dS for AMR determinants for β-lactams, fluoroquinolones, and TMP in 30 *S. suis* isolates.

Gene	Estimated dN/dS (CI)	Selected sites
***pbp1a***	0.13 (0.10-0.18)	6 NSSs
***pbp2b***	0.08 (0.07-0.10)	25 NSSs
***pbp2x***	0.10 (0.08-0.11)	1 PSSs 129 NSSs
***mraY***	0.05 (0.03-0.06)	12 NSSs
***gyrA***	0.03 (0.02-0.05)	21 NSSs
***parC***	0.04 (0.03-0.05)	93 NSSs
***dhfr***	0.19 (0.16-0.22)	13 NSSs

The genes analyzed, the estimated dN/dS (including the 95% confidence interval), and the number of negatively selected codon sites (NSSs) and positively selected codon sites (PSSs) with a significance level of 0.05 are indicated.

CI, confidence interval.

#### 3.4.3 Amphenicols

Amphenicol resistance can be caused by rRNA 23S modification (*cfr, optrA*), enzymatic inactivation of amphenicols (*catA*), and active efflux (*fexA*). The *cfr* gene was not detected in our population. The *optrA* gene was detected in the florfenicol-resistant isolate and in a florfenicol-susceptible isolate out of 115. The *fexA* gene was detected in one florfenicol-susceptible isolate (Ss_49), which also carried the *optrA* gene. PCR analysis in Ss_49 evidenced that both genes were located in tandem on the chromosome, and sequencing analysis revealed an intact *optrA* gene. Thus, apparently, the resistance to florfenicol could be determined by *optrA* (*p*=0.003), but for unknown reasons, its function is lacking in isolate Ss_49.

#### 3.4.4 Fluoroquinolones

Fluoroquinolone resistance is determined by mutations in genes involved in DNA replication, including *gyrA*, *gyrB*, *parC*, and *parE* (Dechêne-Tempier et al., 2021; Uruén et al., 2022). Particularly in *S. suis*, fluoroquinolone resistance is caused by specific mutations in GyrA (S81 and E85) and ParC (S79) (Hadjirin et al., 2021). Hence, we searched for these mutations in 33 *S. suis* isolates with a different AMR profile to fluoroquinolones. Pre-established resistance mutations were detected in isolates Ss_09, Ss_166, Ss_168, and Ss_170 which were resistant to enrofloxacin and marbofloxacin (**Supplementary Figures S3B, C**) but lacked fluoroquinolone-susceptible isolates. Yet, isolate Ss_80, which manifested resistance to marbofloxacin and enrofloxacin, and isolates Ss_02, Ss_21, Ss_22, Ss_24, Ss_45, Ss_48, Ss_70, Ss_72, Ss_84, Ss_92, Ss_100, Ss_106, Ss_115, and Ss_121, which were only resistant to marbofloxacin, lacked the cited mutations. In addition, the amino acid sequences of GyrB and ParE were analyzed in 10 isolates (3 susceptible to both antibiotics, 5 resistant to marbofloxacin, and 2 resistant to both antibiotics). A total of 4 amino acid substitutions were identified in GyrB in all isolates (E219D, V221I, L409F, and A427T), but two of them (E219D and V221I) were only in isolate Ss_166 that manifested resistance to both antibiotics. Besides, a total of 13 amino acid substitutions were detected in ParE, but no one was associated with fluoroquinolone resistance. The dN/dS values for *gyrA* and *parC* were 0.03 and 0.04 (**Table 2**), respectively, indicating they are highly conserved genes. Surprisingly, estimates of recombination revealed multiple recombination events (**Supplementary Table S3**) along their flanking regions (**Supplementary Figure S4**), also suggesting that the acquisition of such specific mutations can be also through horizontal gene transfer. To summarize, resistance to enrofloxacin is mostly attributed to pre-established mutations in GyrA and ParC, but resistance to marbofloxacin was not fully elucidated.

#### 3.4.5 Macrolides, lincosamides and pleuromutilins

Resistance to macrolides and lincosamides is caused by ribosome methylation by sRNA methyltransferases (encoded by *erm* and *cfr* genes), antibiotic modifying enzymes such as phosphotransferases and esterases (encoded by *mph* and *lin* genes), and specific flux pumps (encoded by *mef* or *mrs*(D) genes) (Dechêne-Tempier et al., 2021; Uruén et al., 2022). Only *erm*(B) and *mef*(A/E) were detected in our population. Most of the macrolide-resistant isolates (99%) harbored *erm*(B), and this gene was not present in susceptible isolates. Isolate Ss_80, which manifested tilmicosin-resistance, but was susceptible to tylosin and erythromycin, did not carry *erm*(B) nor *mef*(A/E). Notably, isolates that carried both genes had enhanced MIC values for erythromycin (>2 mg/L), tilmicosin (>64 mg/L), and tylosin (32 mg/L) (*p*<0.001). Interestingly, the genome of the isolate Ss_80 contained an *erm*(47) gene that is linked to erythromycin resistance in other bacteria (**Table 1**), but not reported in *S. suis*. Inspection of the upstream flanking region of the *erm*(47) gene exhibited a large sequence of 67,715 nucleotides that was not present in the public genomes of *S. suis* (**Figure 2**). Blast searchers using this sequence as a query revealed that 55.9% of the nucleotide sequence had >90% of homology with sequences of the genome of *Helcococcus kunzii* strain UCN99 that contains an *erm*(47)-containing element and other AMR genes such as *tet*(T), *sat4*, and *aph*(3’)-IIIa (**Figure 2**). In addition, these regions contain sequences of different sizes that share homology (> 95%) with different bacteria, such as the plasmid pAPRE01 of *Anaerococcus prevotii* DSM 20548, and the genome of *Peptoniphilus* sp. CBA3646, *Amylolactobacillus amylophilus* DSM 20533, *Streptococcus parasuis* SUT-286, and *E. faecalis* isolate 27688. Notably, the upstream and downstream flanking regions of the *erm*(47)-containing element in isolate Ss_80 corresponded to a type I restriction-modification system gene of P1/7 (**Figure 2**). Moreover, the GC content of *erm*(47) and *tet*(T) was 25.9% and 32%, respectively, which deviates from the rest of the genome (42.5%) (Zhang et al., 2011). Together, these data suggest that *erm*(47) and *tet*(T) genes have traveled from another bacterial species to *S. suis* through MGEs.

AMR determinants of lincosamide resistance in *S. suis* are *lnu(*B), *lsa*(E), *oprtA*, *vga*(F) (Dechêne-Tempier et al., 2021; Uruén et al., 2022). *erm*(B) (95.1%), *vga*(F) (20.6%), *lsa*(E) (4.9%), and *lnu*(B) (4.9%) were only present in clindamycin- and lincomycin-resistant isolates in our population (**Table 1**), except for isolate Ss_31 that was susceptible to clindamycin and lincomycin but carried *erm*(B) gene. Sequencing of the full-length *erm*(B) gene in Ss_31 and the adjacent promoter region showed no differences with the homologs in three lincosamide-resistant isolates (data not shown). Statistical analysis revealed that *erm*(B) (*p*<0.001) and *vga*(F) (*p*=0.014) were significantly associated with lincosamide and clindamycin-resistant isolates, but the low prevalence of *lsa*(E) and *lnu*(B) prevented statistical power. Interestingly, lincosamide-resistant isolates with more than 2 AMR genes manifested a higher MIC than 16 mg/L to lincomycin and clindamycin (*p*<0.001). Apparently, accumulation of the cited AMR genes enhances resistance to lincomycin and clindamycin.

Pleuromutilin resistance has been associated with ribosome protection proteins (encoded by *lsa*(E) and *vga*(F)) in *S. suis*. *vga*(F) and *lsa*(E) were present in 21 and 5 out of 25 tiamulin-resistant isolates, respectively, and they were missing in tiamulin-susceptible isolates, except for isolate Ss_52 which contained *vga*(F). Ss_52 manifested a MIC value of 8 mg/L, just below the established breakpoint (**Figure 1**). Statistical analysis showed that both genes, *vga*(F) and *lsa*(E), were significantly related to tiamulin resistance (*p*<0.001). Notably, 2 isolates (Ss_48 and Ss_147) that manifest the highest MIC value (>64 mg/L) carried both genes.

#### 3.4.6 Sulfonamides/TMP

It has been reported that TMP resistance is caused by enzymatic modification (encoded by *dfrF, dfrK*) of the dihydropteroate synthase (encoded by *dhfr*), and a particular mutation in *dhfr* gene (I102L) or its promoter (A5G) (Hadjirin et al., 2021). *dfrF* and *dfrK* genes were not detected in our isolates. Then, the sequence of *dhfr* was analyzed in 35 isolates, including 12 sulfamethoxazole/TMP-resistant (Ss_45, Ss_52, Ss_53, Ss_70, Ss_82, Ss_85, Ss_104, Ss_106, Ss_107, Ss_109, Ss_134, and Ss_138) and 23 sulfamethoxazole/TMP-susceptible isolates (Ss_02, Ss_08, Ss_20, Ss_21, Ss_22, Ss_24, Ss_46, Ss_48, Ss_51, Ss_69, Ss_72, Ss_80, Ss_84, Ss_92, Ss_93, Ss_100, Ss_115, Ss_121, Ss_124, Ss_156, Ss_166, Ss_167, and Ss_168). Amino acidic alteration I102L in *dhfr* gene was identified in all sulfamethoxazole/TMP-resistant isolates and three susceptible isolates (Ss_08, Ss_100, and Ss_166) (**Supplementary Figure S3D**). Several additional mutations were found in sulfamethoxazole/TMP-susceptible and resistant isolates, but none were attributed to resistant isolates. Curiously, isolates Ss_08, Ss_100, and Ss_166 manifested MIC values of 2 mg/L (Ss_08 and Ss_100) or 1 mg/L (Ss_166), just below the pre-established breakpoint (**Figure 1**). The dN/dS value for *dhfr* gene was 0.18 indicating that the gene is indeed conserved (**Table 2**), but several points of recombination (**Supplementary Table S3**) were estimated in the intergenic region located upstream of *dhfr* gene and in a few downstream genes (**Supplementary Figure S4**).

#### 3.4.7 Tetracyclines

Tetracycline resistance in *S. suis* has been related to the presence of efflux pumps (encoded by *tet*(B, K, L, 40)) and ribosome protection proteins (encoded by *tet*(M, O, S, W, 44)). We only detected *tet*(O), *tet*(M), *tet*(W), and *tet*(O/W/32/O) in our population. All genes were detected only in tetracycline- and doxycycline-resistant isolates. *tet*(O) prevailed (89% of resistant isolates), followed by *tet*(M) (6.4%), *tet*(W) (2.7%), and *tet*(O/W/32/O) (2.7%) (**Table 1**). Statistical analysis revealed that *tet*(O) was significantly associated with tetracycline and doxycycline resistance (*p*<0.001), but the low prevalence of the remaining genes and the reduced number of susceptible isolates prevented statistical associations for the remaining AMR genes. However, the presence of 2 AMR genes was significantly related to higher MIC values for doxycycline and tetracycline (*p*<0.001). Isolate Ss_80 was resistant to tetracycline but did not carry any of the cited AMR genes. Whole genome sequence analysis of Ss_80 evidenced the presence of *tet*(T) gene located on the chromosome close to *erm*(47) (**Figure 2**). *tet*(T) gene was not previously reported in *S. suis* but was associated with tetracycline resistance in *S. pyogenes* (Clermont et al., 1997).

### 3.5 Location of AMR genes in MGEs

**Figure 4:**
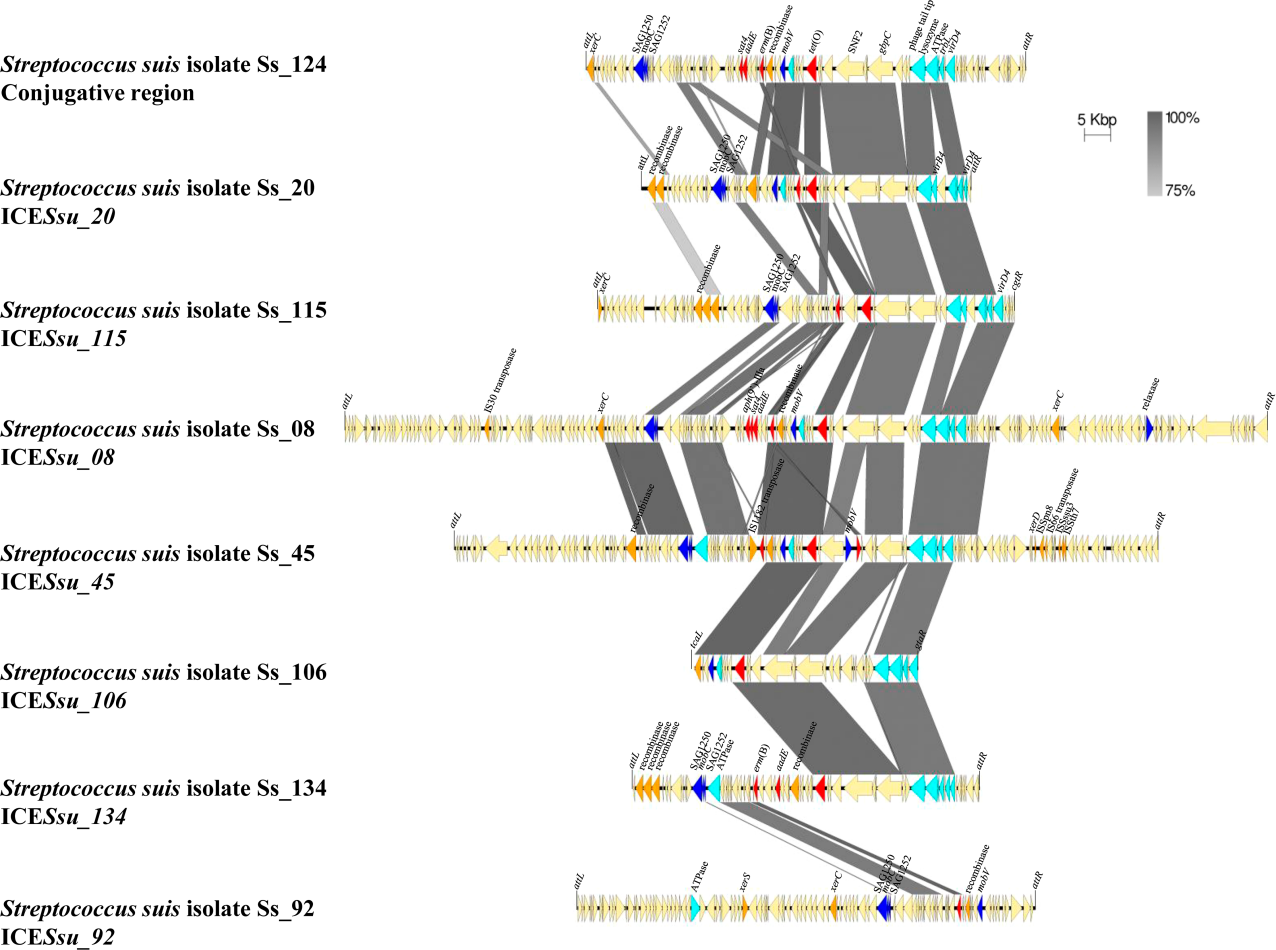
Comparison of the genetic organization of eight MGEs detected in *S. suis* isolates. Nucleotide sequences of 1 conjugative region and 7 ICEs carrying AMR genes are compared. Light blue arrows indicate genes coding for Type IV Secretion System, dark blue arrows indicate gene coding for relaxases, orange arrows indicate genes coding for integrases, and red arrows indicate AMR genes. Integration sites are shown.

The 30 genomes of our isolate collection were used for detecting MGEs, analyzing their structure, and if they transfer AMR genes. A total of 35 ICEs, 20 IMEs, and 2 conjugative elements were identified in the 30 genomes of the invasive isolates analyzed here (**Supplementary Table S4**). 18 out of 60 of these MGEs carried at least one or more AMR genes, including *aph*(3´)-IIIa, *sat4, aadE, erm*(B), *tet*(O), and *tet*(M). Notably, all of them carried at least *erm*(B) and/or *tet*(O), except Ss_51, which only carried *tet*(M). Ss_08 carried an ICE harboring 5 AMR genes (*aph*(3’)-IIIa, *sat4*, *aadE*, *erm*(B), and *tet*(O)). MGEs had different gene content and length, but shared regions of different size (**Figure 4**), mainly the upstream region that harbors genes coding for Type IV Secretion System. However, downstream regions were shared between MGEs. This evidence the occurrence of many recombination events between MGEs resulting in the gain or loss of genes, and thus explains the large repertoire of MGEs and the variable combinations of AMR genes (**Figure 2**). Moreover, identical IMEs were identified in isolates of the same genetic background. Examples included isolates Ss_100, Ss_106, and Ss_156 of ST123 that contained the same IME of 5,994 bp. These isolates were recovered in neighbor Autonomous Communities (*i.e.*, Aragón and Cataluña). But, curiously, isolates Ss_20 and Ss_92 of ST29 contained a similar 5,987 bp IME but were recovered from geographically distant Autonomous Communities (*i.e.*, Aragón and Extremadura). Anyhow, these data demonstrate that some AMR genes are located in MGEs which may serve for their transference between *S. suis* isolates. The presence of multiple AMR genes in MGEs may explain the rapid occurrence of multi-drug resistance.

### 3.6 Dissemination of AMR genes of S. suis among pathogenic bacteria

**Figure 5:**
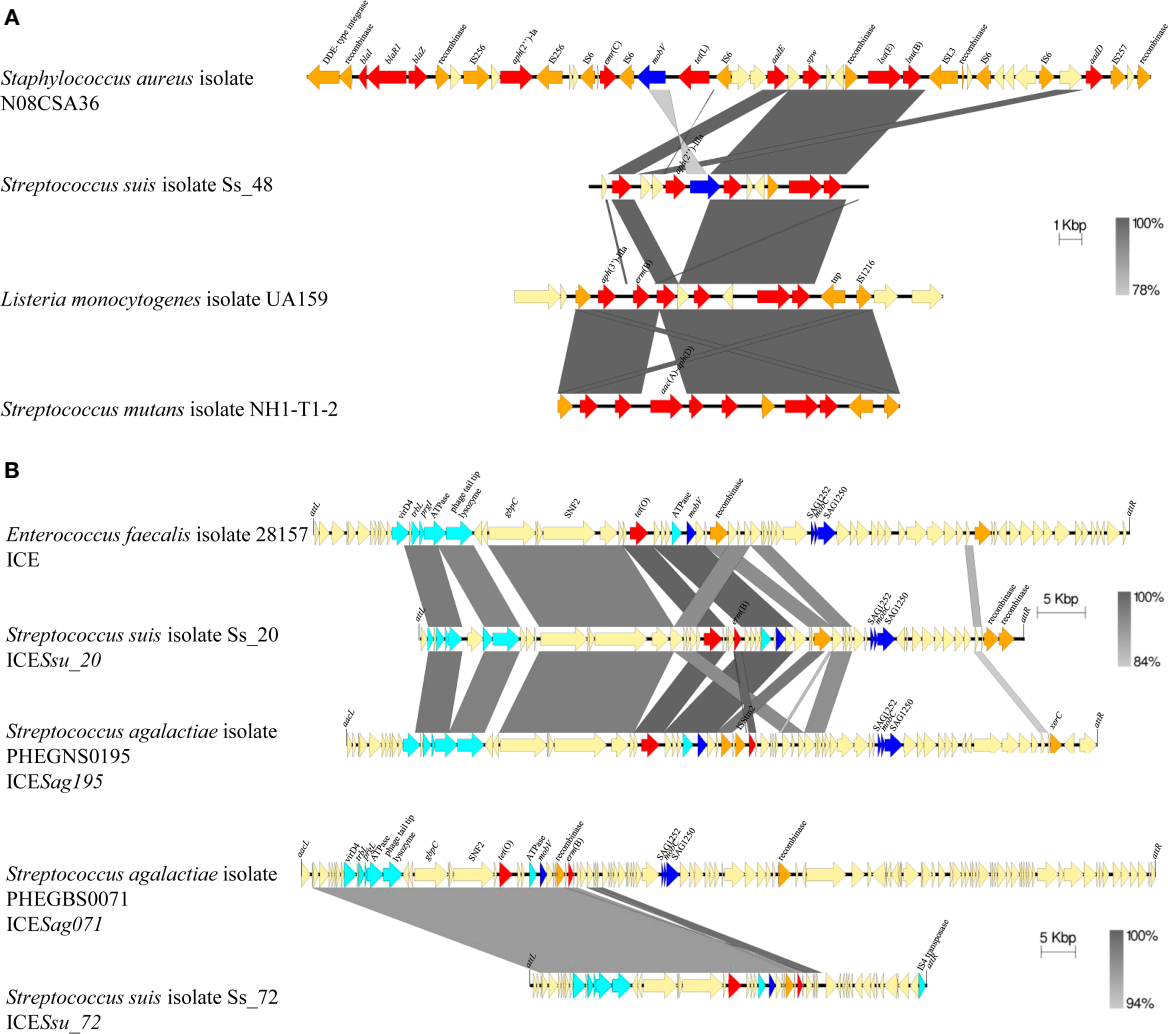
Comparison of the genetic organization of *S. suis* isolates with genomes of diverse pathogenic bacteria. **(A)** Comparison of genomic islands carrying AMR genes. **(B)** Comparison of ICEs carrying AMR genes. Light blue arrows indicate genes coding for Type IV Secretion System, dark blue arrows indicate gene coding for relaxases, orange arrows indicate genes coding for integrases, and red arrows indicate AMR genes. Integration site for ICEs is indicated.

The GC content of several AMR genes present in our *S. suis* genomes, including *erm*(47) (25.9%), *lnu*(B) (31.1%), *tet*(T) (32%), *lsa*(E) (32.4%), *aadE* (32.9%) and *erm*(B) (33.1%), *apmA* (44.6%), and *aph*(3’)-IIIa (44.9%), deviates from the rest of genome (41.2%) (Zhang et al., 2011). This suggests that many of them were acquired from another bacterium. We performed nucleotide BLAST searches to investigate their dispersion in public genomes. A summary of major hits is listed in **Table 1**. For example, when using *spw* gene of the isolate Ss_48 as a query in BLAST, we identified homologs in many genomes of different pathogenic and non-pathogenic bacteria, including 33 *Enterococcus faecium*, 24 *E. faecalis*, 9 *S. aureus*, 7 *Erysipelothrix rhusiopathiae, 4 Clostridioides difficile*, diverse streptococci species (3 *Streptococcus agalactiae*, 1 *Stretptococcus dysgalactiae*, 2 *Streptococcus mutans*, and 1 *Streptococcus parasuis*), and 1 *Listeria monocytogenes*. In the genome of *L. monocytogenese*s UA159, the *spw* homolog is located in a genomic island together with other AMR genes (*aph*(3’)-IIIa, *aadE*, *spw*, *lnu*(B), and *lsa*(E)) flanked by genes coding for two transposases (**Figure 5A**). Notably, *aadE*, *spw*, *lnu*(B) and *lsA*(E) of *L. monocytogenesi*s UA159 are homologs (100% identity) to those of *S. suis* isolate Ss_48 although the genetic organization varied. But, also homologs (100% identify) of the *S. suis aadE* gene were identified in 25 genomes of *E. faecium*, 21 of *E. faecalis*, 3 of *E. rhusiopathiae*, 20 of *S. aureus*, 4 of *C. difficile, 1* of *L. monocytogenes*, and various streptococci species (3 *S. agalactiae*, 1 *S. dysgalactiae*, and 2 *S. parasuis*) (**Table 1**). However, their genomic organization varied among genomes. For example, in the genome of *S. aureus* strain N08CSA36, the *aadE* gene is located together with 9 additional AMR genes (*blaI*, *blaR1*, *blaZ*, *aph*(2’’)-Ia, *erm*(C), *tet*(L), *spw*, *lsa*(E), and *lnu*(B)), and genes coding for diverse transposases, relaxases and a *mobV* recombinase, the latter is also in *S. suis* Ss_48 (**Figure 5A**). Also, a homolog (100% identity) of the *lsa*(E) gene of isolate Ss_48 was detected in *S. mutans* strain NH1-T1-2, which was located together with *aadE*, *spw*, and *lnu*(B) genes and a recombinase (**Figure 5A**). The presence of shared regions between genomes, genes coding for recombinases and transposases and several insertion sequences may contribute to the allelic exchange and the gain or loss of AMR genes.

Homologs of the *erm*(B) gene of isolate Ss_08 were also identified in 67 of *E. faecium*, 8 of *E. faecalis*, 1 of *Campylobacter coli*, 1 of *S. agalactiae*, and 1 of *Streptococcus mitis*. Also, homologs of *tet*(O) of *S. suis* isolate Ss_08 were detected in 25 genomes of *S. agalactiae*, 2 of *S. pneumoniae*, 1 of *S. dysgalactiae*, and 1 of *E. faecalis* with 100% identity, and *tet*(O) variants (>99% identity) in 3 genomes of *Campylobacter jejuni*, 2 of *S. agalactiae*, 2 of *S. mutants*, 1 of *Staphylococcus coagulans*, 1 of *S. dysgalactiae*, 1 of *Salmonella enterica* and 1 of *Clostridium* sp. (**Table 1**). In *S. suis* Ss_08 *tet*(O) is placed together with *erm*(B) in an ICE. A sequence of 17,306 bp shares a 50-98% coverage with more than 94% of identity with genomes of 42 bacterial species, such as *S. agalactiae* S5 (86% coverage, 94.45% identity), *S. pyogenes* strain TSPY453 (59% coverage, 94.6% identity), *S. dysgalactiae* strain DY107 (91% coverage, 95.5% identity), *E. faecalis* isolate 28157_4#173 (72% coverage, 99.9% identity), and several species of *Clostridium* including *C. scindens* strain BL389WT3D (75% coverage, 94.6% identity). Also, in *S. suis* isolate Ss_20, *tet*(O) and *erm*(B) genes are located in an ICE, which shares 37,584 bp and 37,979 bp (>90% identity) with ICEs placed in *E*. *faecalis* isolate 28157 and *S. agalactiae* isolate PHEGNS0195 (**Figure 5B**). Also, *S. suis* isolate Ss_72 contains an ICE with *tet*(O) and *erm*(B) genes in a different genomic organization but that share 41,726 bp (> 96% homology) with an ICE of *S. agalactiae* isolate PHEGBS0071 (**Figure 5B**) and 6807 bp (44-100% coverage, 84-100% identity) with the plasmid pELF_mdr in strain NUITM-VRE1 of *E. faecium* (52% of coverage, 99.6% identity), the plasmid pRE25 in *E. faecalis* strain RE25 (54% of coverage, 99.9% identity), genomes of 25 species, including *S. agalactiae* strain FDAARGOS_670 (100% coverage, 99.9% identity), *S. mutans* strain NH1-T1-1 (52% coverage, 99.8% identity), *S. aureus* strain AOUC-0915 (54% coverage, 96.4% identity), *S. coagulans* strain 1031373 (68% coverage, 95.8% identity), *C. difficile* strain CD161 (48% coverage, 95.6% identity), among others. In contrast to the cited AMR genes, homologs of *vga*(F), which GC content was similar to *S. suis* genome, were only identified in streptococci species (**Table 1**). In conclusion, several AMR genes identified in Spanish *S. suis* have a different phylogenetic origin and seem to be mobilized through different genetic mechanisms.

## 4 Discussion

High AMR rates for lincosamides, macrolides, and tetracyclines in *S. suis* have been reported globally (Uruén et al., 2020). A recent study testing 103 *S. suis* isolates from Spain revealed AMR rates for clindamycin and tylosin up to 87% (Petrocchi-Rilo et al., 2021), which confirms our results. Both antibiotics were used in pig production to treat a diversity of bacterial infectious diseases in Spain. Because *S. suis* is a commensal, the high AMR rates found here could be caused by its exposition to the cited antibiotics. Lincosamides and macrolides often show cross-resistance caused by mutual resistant mechanisms. The AMR for erythromycin and lincosamides found in this work were attributed to *erm*(B) which exhibited a high prevalence (>98%) in resistant isolates. *erm*(B) encodes for a methylase that modifies ribosomal 23S rRNA, often located in MGEs (Huang et al., 2016; Chen et al., 2021). Notably, we identified *erm*(B) located together with *tet*(O). This genetic linkage between both genes was early reported (Huang et al., 2016). The co-localization of both genes in MGEs could contribute to the high co-occurrence of erythromycin and tetracycline resistance.

The AMR rate for enrofloxacin (30%) was similar to that reported recently in Spain (Petrocchi-Rilo et al., 2021), but much higher than an earlier work (2005) (Vela et al., 2005), suggestive of an enhancement of AMR in the last decade. This rate is considerably higher than other European countries such as France (18%) (Vachee et al., 2008), Belgium (0.3%) (Callens et al., 2013), The Netherlands (0.6%) (van Hout et al., 2016), or Sweden (5.3%) (Werinder et al., 2020). Enrofloxacin-resistance was mainly caused by particular mutations in pre-established quinolone-resistance determining region of *gyrA* and *parC* genes as reported (Escudero et al., 2007), however, they do not fully explain marbofloxacin-resistance. Five marbofloxacin-resistant but enrofloxacin-susceptible isolates lacked pre-established genotypes. Also, mutations in *gyrB* and *parE* can contribute to enrofloxacin resistance in streptococci (González et al., 1998; Jorgensen et al., 1999). Sequencing of *gyrB* and *parE* in our isolates did not show differences between susceptible and resistant isolates. Alternatively, efflux pumps such as PmrA in *S. pneumoniae* (Gill et al., 1999) cause fluoroquinolone resistance, but no homologous were found in our genomes.

Tiamulin is broadly used to treat *S. suis* infections. The AMR rate for tiamulin (19%) matches that recently reported (12%) in Spain (Petrocchi-Rilo et al., 2021) and in some European countries such as Denmark (2020) (20%) (DANMAP, 2021) or England (2014) (23%) (Hernandez-Garcia et al., 2017). Notably, Thailand reported rates up to 80% (2019) (Yongkiettrakul et al., 2019). Tiamulin resistance was caused by *vga*(F) and *lsa*(E) genes, which code for ABC-F proteins with a function as ribosome-protection proteins. Hence, they can confer resistance to different ribosome-targeting antibiotics such as lincosamides and pleuromutilins (Uruén et al., 2020), as we find here.

The AMR rate for florfenicol (5%) was considerably lower than that recently reported (Petrocchi-Rilo et al., 2021) (14%), but in line with many European countries such as Denmark (2020) (0%) (Arndt et al., 2019) or The Netherlands (van Hout et al., 2016) (0.1%). Also, low resistance rates were reported in American countries such as Canada (2019) (0.5%) (Arndt et al., 2019) or USA (2016) (1%) (Hayer et al., 2020). The only florfenicol-resistant isolate contained an *oprtA* gene that encodes an ABC-F family protein producing ribosome protection. *oprtA* gen is often reported inside of MGEs (Huang et al., 2017; Shang et al., 2019; Zhang et al., 2020), sometimes together with other AMR genes. Actually, it was reported in *S. suis* isolates in China with a prevalence ranging from 11%-38% (Huang et al., 2019; Shang et al., 2019; Zhang et al., 2020). Also, we detected the *fexA* gene that codes for an efflux pump that exports amphenicols, but it could not be related to florfenicol resistance. Surprisingly, previous studies demonstrated a high prevalence of *fexA* (26%) in Spanish *S. suis* isolates by PCR screening (Petrocchi-Rilo et al., 2021), but its presence could not be related to chloramphenicol resistance. When we used primers described by the authors to detect *fexA*, an amplicon was produced in several isolates but sequencing revealed sequences of a putative transketolase-subunit. New primers were designed here, and all resulting amplicons were sequenced. We hypothesized that the prevalence of *fexA* previously reported in Spain is overestimated.

β-lactams are the gold-standard treatments against *S. suis* disease. AMR rates for β-lactams varied considerably among antibiotics being high for penicillin (32%). Low resistance rates of β-lactams (<10%) were reported in other European countries such as The Netherlands (van Hout et al., 2016) or The Czech Republic (Matiašovic et al., 2021). This huge difference could be caused by their extensive use in Spain, sometimes as metaphylactic therapies. Importantly, our statistical analysis revealed an enhancement of penicillin G-resistance in a 6-year period. Earlier work in Spain (2005) reported low AMR rates for penicillin (4%) and amoxicillin (0.7%) in 151 clinical isolates (Vela et al., 2005). Recent work (Petrocchi-Rilo et al., 2021) reported a low resistance to ampicillin (3%), and higher to ceftiofur (17%) and penicillin (26%). Altogether, it seems penicillin resistance is rapidly increasing in Spain. Notably, the low AMR rates in ST1 isolates and high AMR rates in ST123 isolates are in agreement with studies in other countries (Cucco et al., 2022).

We detected a high amino acid diversity in PBPs in penicillin-resistant compared to susceptible isolates in order PBP2X > PBP2B > PBP1A. Previous reports showed that mutations in PBP2B and PBP2X confer moderate AMR, while in combination with mutations in PBP1A confer high AMR (Hadjirin et al., 2021). It has been postulated in other streptococci species that such mutations are acquired by interspecies gene transfer (Hakenbeck et al., 2012). Our bioinformatics analysis is in line with this hypothesis, as we found significant shreds of evidence of recombination within *pbp* genes. Furthermore, we detected particular mutations, only present or, highly associated with penicillin-resistant isolates; some were previously detected by other authors, for example, K479T, D512E in PBP2B or T551S in PBP2X (Hadjirin et al., 2021), but many others were not reported before (Bamphensin et al., 2021; Hadjirin et al., 2021; Lunha et al., 2023). Hence, penicillin resistance can be acquired by one or a combination of mutations that probably affect the binding of the enzyme to penicillin. However, the estimates of dN/dS for these genes indicated a strong negative selection for amino acid replacement. This matches their relevant role in bacterial growth. Therefore, genetic transference enables penicillin resistance, but the accumulation of mutations probably alters the properties and function of the enzymes compromising bacterial survival. Thus, only mutations that balance bacteria survival and antibiotic resistance are selected. Moreover, mutations in MraY have been related to β-lactam resistance in *S. suis* (Hadjirin et al., 2021). MraY transfers N-acetylmuramyl-pentapeptide-1phosphate to undecaprenyl phosphate to generate the peptidoglycan precursor. MraY is not a target for β-lactams, probably these mutations are compensatory for those in PBPs, and thus they improve bacterial fitness. Also, during the recombination process, adjacent genes to *pbp*s may be affected, including *ddlA*, that form part of the biosynthesis of the peptidoglycan precursor, or *omsC* that encodes for a putative osmotically inducible protein, among others (**Supplementary Figure S4**). Their contribution to AMR or bacterial fitness should be elucidated in future studies.

To the best of our knowledge, we identified four novel AMR genes in *S. suis*. *tet*(T) gene encodes for a ribosomal-protection protein. They were identified in other streptococci (Clermont et al., 1997; Stefańska et al., 2022), or enterococci (Nishimoto et al., 2005). *ampA* gene encodes for an N-acetyltransferase to N2´of apramycin (Bordeleau et al., 2021). Apramycin is an antibiotic broadly used in veterinary medicine for decades. *ampA* gene was identified in diverse pathogens including *S. aureus (* Feßler et al., 2011) and *Campylobacter* (Fabre et al., 2018). It is often located on plasmids together with other AMR genes (Feßler et al., 2018) that favor its dissemination. Finally, *erm*(47) encodes for a protein sharing 44%-48% amino acid identified with Erythromycin methylases in *Helcococcus kunzii (* Guérin et al., 2016). *H. kunzii* is part of the skin microbiota of pigs and can become an opportunistic pathogen (Grattarola et al., 2010). Besides, *aph*(2’’)-IIIa is present in a variety of bacterial species including *Enterococcus*, *Staphylococcus*, or *Campylobacter*, among others. Also, blast searchers with several AMR genes identified homologs in genomes of many other bacteria, including human and animal pathogens. Some genes are located in MGEs which can facilitate horizontal gene transfer through conjugation. Our analysis evidenced that MGEs can contain a large repertoire of AMR genes that favor the rapid acquisition of multi-drug resistance and explain the different multi-drug resistance profiles. Together, our data support that Spanish *S. suis* has acquired AMR genes from other species through multiple and independent events. Considering experimental evidence that *S. suis* can transfer AMR genes to different pathogenic bacteria (Marini et al., 2015; Huang et al., 2016), these data point out that *S. suis* is a relevant contributor to the spread of AMR genes across human and animal pathogens.

## 5 Concluding remarks

To summarize, here we reported the AMR rates for different antibiotics in circulating clinical isolates of *S. suis* in Spain. High rates of resistance were found to lincosamides, tetracyclines, and erythromycin following a global trend. However, our study also reflects the emergence of resistance to enrofloxacin, sulphonamides, and penicillin G in Spain showing higher AMR rates than in studies reported in other European countries. Importantly, multidrug resistance was observed in 90% of the isolates. Indeed, particular actions must be taken to control AMR of *S. suis* in Spain. Additionally, our work also revealed the genetic mechanisms contributing to AMR for most antibiotics, involving genes coding for target-protecting proteins (*optrA*, *erm*(B), *lsa*(E), *vga*(F), *tet*(M), *tet*(O), *tet*(O/W/32/O), *tet*(W), *tet*(T), *aph*(2’’)-IIIa) antibiotic-modifying enzymes (*aph*(3’)-IIIa, *sat4*, *aadE*, *spw*, *aac*(6’)-Ie-*aph*(2’’)-Ia, *mrs*(D), *mph*(C), *lnu*(B), *erm*(47)), active efflux pumps (*fexA*, *mef*(A/E)), and mutations in chromosomal genes (*pbp1a*, *pbp2b*, *pbp2x*, *mraY*, *gyrA*, *parC*, and *dhfr*). Our genetic analysis and comparisons showed evidence for the genetic transference of AMR genes between *S. suis* isolates and strains of other species, which explains the broad AMR dissemination and multidrug resistance of *S. suis*.

## Data availability statement

The datasets presented in this study can be found in online repositories. The names of the repository/repositories and accession number(s) can be found below: Bioproject, PRJNA1037519, Ss_02 (ASM3380320v1), Ss_69 (ASM3380323v1), Ss_70 (ASM3380322v1), Ss_80 (ASM3380318v1), Ss_92 (ASM3380316v1), Ss_93 (ASM3380314v1), Ss_100 (ASM3380312v1), Ss_121 (ASM3380306v1), Ss_156 (ASM3380310v1), Ss_166 (ASM3380308v1), and Ss_167 (ASM3380302v1).

## Author contributions

CU: Data curation, Investigation, Methodology, Software, Visualization, Writing – original draft, Writing – review & editing. JG: Investigation, Writing – review & editing. MS: Investigation, Writing – review & editing. LF: Investigation, Methodology, Supervision, Writing – review & editing. CM: Investigation, Funding acquisition, Writing – review & editing. JA: Funding acquisition, Conceptualization, Project administration, Resources, Supervision, Writing – original draft, Writing – review & editing.

## Supplementary material

Supplementary Table 1
*S. suis* isolates used in this study and the MIC values for 18 antibiotics. The geographic origin, ST, serotype, and host is indicated.

Supplementary Table 2 Primers used in this study.

Supplementary Table 3 Events of genomic recombination detected in 19 genomes of *S. suis*.

Supplementary Table 4 Mobile genetic elements found in isolates of *S. suis* by whole genome sequencing.

Supplementary Figure 1 Maximum-likelihood phylogenetic tree constructed with the nucleotide sequences of the 30 genomes of our *S. suis* collection (colored in black) and 8 reference genomes (colored in red). The genetic characteristics of serotype, ST, eBG and CC are given in the first 4 columns (color code as indicated in the figure legend). From column 5 onwards, the resistance profile to the 17 antimicrobials tested is shown in green when the isolate is susceptible, red when is resistant, and yellow when it has intermedia resistance. ST, Sequence Type; eBG, eBurst Group; CC, Clonal Complex; ser, Serotype; SPE, Spectinomycin; AMOX, Amoxicillin; AMP, Ampicillin; CEFQ, Cefquinome; CEFT, Ceftiofur; PG, Penicillin G; FLO, Florfenicol; ENR, Enrofloxacin; MAR, Marbofloxacin; CLI, Clindamycin; LIN, Lincomycin; ERY, Erythromycin; TIL, Tilmicosin; TYL, Tylosin; TIA, Tiamulin; S/TMP, Sulfamethoxazole/Trimethoprim; DOX, Doxycycline; TET, Tetracycline.

Supplementary Figure 2 Distribution of multi-resistance patterns identified in 116 invasive *S. suis* isolates. The number of antibiotic families and the percentage of isolates in each pattern is indicated.

Supplementary Figure 3 Alignment of sequences of AMR determinants in susceptible and resistant isolates. Alignments comprise **(A)** *spw* gene and the upstream sequence of one spectinomycin resistant and three spectinomycin-susceptible isolates, **(B)** GyrA sequences of fluoroquinolone resistant and susceptible isolates, **(C)** ParC of fluoroquinolone resistant and susceptible isolates, and **(D)** DHFR of sulfamethoxazole/TMP resistant and susceptible isolates. In panels b-d, the site position previously associated with antibiotic resistance are indicated, including positions 81 and 85 for GyrA, position 79 and 83 for ParC, and 102 position for DHFR. The sequence of the reference genome P1/7, which is susceptible to all antibiotics, is included. Susceptible isolates are green coloured, resistant are red coloured, and, in the case of fluoroquinolones, marbofloxacin resistant isolates are orange coloured otherwise indicated.

Supplementary Figure 4 Genetic organization of different genes involved in AMR. The shadow indicates sequences where recombination breakpoints were estimated. HP, Hypothetical protein; ABC-t, ABC transporter ATP-binding protein.
