# Supplementary material for: Invasive Streptococcus suis isolated in Spain contain a highly promiscuous and dynamic resistome
Source: Front Cell Infect Microbiol. 2024 Jan 22;13:1329632. doi: 10.3389/fcimb.2023.1329632 (PMC10839070; doi:10.3389/fcimb.2023.1329632)
Supplement: Supplementary Table 2 — Primers used in this study. [file Table_2.pdf]

Table S2. Primers used in this study. Sequence, temperature of annealing (T_A), expected product size and reference are indicated.

Primer	Forward (3'-5')	Reverse (3'-5')	T_A	Product size (bp)	Putative gene	Reference
**Detection/amplification of AMR genes**						
aphA3	GGCTAAAATGAGAATATCACCGG	CTTTAAAAAATCATACAGCTCGCG	47	526	*aph*(3')-IIIa	(Vakulenko et al., 2003)
ant1	AATTTCTGGCTCCGTAGGCTG	CCCATTTTGCGGCAACATCT		410	*ant1*	This study
aacA	TAATCCAAGAGCAATAAGGGC	GCCACACTATCATAACCACTA	55	228	*aacA*	(Strommenger et al., 2003)
aadE	TAAGTGATATAGAACCGTTTAT	CAAATATTTTCATAGGAATCC	44	568	*aadE*	This study
ant9	CGGTCAGCTTTATTGAACAG	TAATTTCACAAGAGGACGCT	55	989	*ant*(9')-Ia	(Mahbub Alam 2005)
aac6-aph2	CAGAGCCTTGGGAAGATGAAG	CCTCGTGTAATTCATGTTCTGGC	55	348	*aac*(6')-Ie-*aph*(2'')	(Vakulenko et al., 2003)
sat4	AAAACCGGCTGCCTGGATAG	TCGACGGAGCCGATTTTGAA	55	532	*sat4*	This study
spw	GGCAGTAATGGGTGGTTTACG	CCCATTCAGCTGCCAGATCT	55	521	*spw*	This study
apmA	CGCGAGGTAGTTGTATGGGG	TATAGCACCGTCACCGATGC	55	567	*apmA*	This study
aph2	CAAAAGATCGACTGGCGCTG	TCGCACCAAAATTGTTGGCC	55	576	*aph*(2'')-IIIa	This study
pbp2x	ATACGACAATTTCAGCCGAT	TTAATTTCAGCACCGTTTCC	55	1219	*pbp2x*	This study
pbp2b	GCTGATTTAGCCTTTCAAGATG	AGCCTCCTTAATTCATTGGG	55	1127	*pbp2b*	This study
pbp1a	GCGTGGAATATCTACAATACAGA	ACGATTGGTATATCCGGTCC	55	894	*pbp1a*	This study
mraY	CGTTTGAATAACCACTCCATAT	TCGGTACCGCTTTGAATAAA	55	1191	*mraY*	This study
fexA	GCGTCAATCCCGTACTTCCACT	CTGCGGCGTTATTTGCGGGA	55	1065	*fexA*	This study
optrA	AGGTGGTCAGCGAACTAA	ATCAACTGTTCCCATTCA	55	1395	*optrA*	(Wang et al., 2015)
gyrA	AGGGGATGTTATGGGTAAAT	GTGAATTCCAGATTTCCCCAT	55	481	*gyrA*	This study
parC	CTTGAGGACATCATGGGAGAG	TTCCGTATCGTCAAAGTTCC	55	423	*parC*	This study
ermB	GCAGTGACTAATCTTATGACTTTT	CTGTGGTATGGCGGGTAAGT	63	621	*erm*(B)	(Malhotra-Kumar et al., 2005)
mefA-E	CAATATGGGCAGGGCAAG	AAGCTGTTCCAATGCTACGG	62	315	*mef*(A/E)	(Malhotra-Kumar et al., 2005)
mrsD	CCTTATCGGCACAGGTTCAT	GAATGCCTTCCGGAGCTTC		489	*mrs*(D)	(Lüthje et al., 2007)
erm47	GTGAATTCTAATGATGTTTA	ATGGATAGAATTTGATCAA	55	623	*erm*(47)	This study
mphB	ATTAAACAAGTAATCGAGATAGC	TTTGCCATCTGCTCATATTCC	50	869	*mph*(B)	(Achard et al., 2008)
mphC	GAGACTACCAAGAAGACCTGACG	CATACGCCGATTCTCCTGAT	59	722	*mph*(C)	(Lüthje et al., 2007)
cfr	TGAAGTATAAAGCAGGTTGGGAGTCA	ACCATATAATTGACCACAAGCAGC	48	748	*cfr*	(Kehrenberg et al., 2006)
lnuB	CGTGGGGAATTTCATTTCCTTTC	CGTTGATTCCCATCAACCATAG	56	226	*lnu*(B)	(Bojarska et al., 2016)
lsaE	TATGCGTATTCCGGCAATATAAG	AACGGCTTCCTGATGTCTTG	56	681	*lsa*(E)	(Bojarska et al., 2016)
vgaF	TGTATTCAATTAGAGAAGGA	GATTTTCAAAAAAGTAGACT	55	129	*vga*(F)	This study
dhfr	CCTACTTGGTAAGTGCATTTT	GCGTAGGTTTTTATTGTAAAATCA	55	558	*dhfr*	This study
tetO	AACTTAGGCATTCTGGCTCAC	TCCCACTGTTCCATATCGTCA	61	515	*tet*(O)	(Malhotra-Kumar et al., 2005)
tetM	AGAACTCGAACAAGAGGAAA	ATGGAAGCCCAGAAAGGAT	60	471	*tet*(M)	(Olsvik et al., 1995)
tetT	AATCTTGCAATAATGCAATCA	TGGATTACTGCTTTAGAGTTTC	55	869	*tet*(T)	This study
tetL	TGGTGGAATGATAGCCCATT	CAGGAATGACAGCACGCTAA	62	229	*tet*(L)	(Malhotra-Kumar et al., 2005)
tetK	GATCAATTGTAGCTTTAGGTGAAGG	TTTTGTTGATTTACCAGGTACCATT	62	155	*tet*(K)	(Malhotra-Kumar et al., 2005)
tetW	GGAGGAAAATACCGACATA	AATCTTACAGTCCGTTACG	60	1000	*tet*(W)	(Patterson et al., 2007)
tet40	CGGAGGAAGAGGACAAACCC	TAAGCCGCTGCCGATAAGAC	56	446	*tet*(40)	(Chen et al., 2013)
tet44	GGTATTCTTGCTCATGTAG	TCCATGGTATATAGGAAACA		639	*tet*(44)	This study

tetW-Fw / tetM-Rev*[2]	GGAGGAAAATACCGACATA	ATGGAAGCCCAGAAAGGAT	60	834	*tet*(O/W/32/O)	(Olsvik et al., 1995)
**External to AMR genes**						
spw_EXT	GGTGGCTCAAACATATAATAAAC	GAGTTGATGGCATTGATGT	55	1112	*spw*	This study
pbp2x-mraY_EXT	AGAGATTGATGGGATTTGTTATGATTT	CGACAAATTCGCCATTTTTCATTT	55	6373	*pbp2x-mraY*	This study
pbp1a_EXT	TTCCCCAGCATCCCATATCT	CGCTTTCCTCGGTAATAGCTG	55	2435	*pbp1a*	This study
pbp2b_EXT	TCTGTCTAAAAAATATTTTTTGCCT	pbp2b-Rev	55	2150	*pbp2b*	This study
fexA-optrA_EXT	ATGAAAAAGGATAGTAAATC	GCGATTATATCTAATAAAAATTC	55	4176	*fexA-optrA*	This study
gyrA_EXT	ATTGACTGGACGAACGATAC	GGCGATAATTATCTACCAAAGC	55	5494	*gyrA*	This study
ermB_EXT	GCGCAATCTTATGACTTTTTAAACAGA	AGTAACGTGTAACTTTCCAAATTT	55	876	*erm*(B)	This study
**Sequencing**						
pbp2x_Seq	TTATGCAAACGCGGCTTTT		-	-	*pbp2x*	This study
pbp2x-mraY_Seq1	CAATAATCGTGCCCATTC	GCAAGTCTTTTGGCAGGC	-	-	*pbp2x-mraY*	This study
pbp2x-mraY_Seq2	AATGGAAATTTTGCTTCA	GAAAATCTTCGCTGGTTT	-	-	*pbp2x-mraY*	This study
pbp2x-mraY_Seq3	TGGTATGATGTTAGAGCC	GTAGGAGTTCCGGCCTTG	-	-	*pbp2x-mraY*	This study
pbp1a_Seq	CAAGAGATTTTGACCTACT		-	-	*pbp1a*	This study
gyrA_Seq		AACTTCTGGCTCACGCTCAC	-	-	*gyrA*	This study
fexA_Seq	TCAATAACAAGGATTATG	CAGCAGTTCCAACACCAA	-	-	*fexA*	This study
optrA_Seq1	GGTGTAAGCTAAAACTTA	ATGTAGATTTATAACTTC	-	-	*optrA*	This study
optrA_Seq2	TTAAAGTTGAGCAGAAAG		-	-	*optrA*	This study

T_A: Annealing temperature, Fw: Forward, Rev: Reverse, EXT: exterior, Seq: Sequencing.

References

Achard, A., Guérin-Faublée, V., Pichereau, V., Villers, C., Leclercq, R. (2008). Emergence of macrolide resistance gene *mph*(B) in *Streptococcus uberis* and cooperative effects with *rdmC*-like gene. *Antimicrob. Agents Chemother*. 52 (8), 2767–2770. doi: 10.1128/AAC.00481-08.

Bojarska, A., Molska, E., Janas, K., Skoczyńska, A., Stefaniuk, E., Hryniewicz, W., et al. (2016). *Streptococcus suis* in invasive human infections in Poland: clonality and determinants of virulence and antimicrobial resistance. *Eur. J. Clin. Microbiol. Infect. Dis.* 35 (6), 917–925. doi: 10.1007/s10096-016-2616-x.

Chen, L., Song, Y., Wei, Z., He, H., Zhang, A., Jin, M. (2013). Antimicrobial susceptibility, tetracycline and erythromycin resistance genes, and multilocus sequence typing of *Streptococcus suis* isolates from diseased pigs in China. *J. Vet. Med. Sci.* 75 (5), 583–587. doi: 10.1292/jvms.12-0279.

Kehrenberg, C., Schwarz, S. (2006). Distribution of florfenicol resistance genes *fexA* and *cfr* among chloramphenicol-resistant *Staphylococcus* isolates. *Antimicrob. Agents Chemother.* 50 (4), 1156–1163. doi: 10.1128/AAC.50.4.1156-1163.2006.

Lüthje, P., Schwarz, S. (2007). Molecular basis of resistance to macrolides and lincosamides among staphylococci and streptococci from various animal sources collected in the resistance monitoring program BfT-GermVet. *Int. J. Antimicrob. Agents*. 29 (5), 528–535. doi: 10.1016/j.ijantimicag.2006.

Mahbub Alam, M., Kobayashi, N., Ishino, M., Sumi, A., Kobayashi, K.I., Uehara, N., et al. (2005). Detection of a novel *aph*(2") allele (*aph*[2"]-Ie) conferring high-level gentamicin resistance and a

spectinomycin resistance gene *ant*(9)-Ia (*aad9*) in clinical isolates of enterococci. *Microb. Drug Resist*. 11 (3), 239–247. doi: 10.1089/mdr.2005.11.239.

Malhotra-Kumar, S., Lammens, C., Piessens, J., Goossens, H. (2005). Multiplex PCR for simultaneous detection of macrolide and tetracycline resistance determinants in streptococci. *Antimicrob. Agents Chemother*. 49 (11), 4798–4800. doi: 10.1128/AAC.49.11.4798-4800.2005.

Olsvik, B., Olsen, I., Tenover, F.C. (1995). Detection of *tet*(M) and *tet*(Q) using the polymerase chain reaction in bacteria isolated from patients with periodontal disease. *Oral Microbiol. Immunol*. 10 (2), 87–92. doi: 10.1111/j.1399-302x.1995.tb00124.x.

Patterson, A.J., Rincon, M.T., Flint, H.J., Scott, K.P. (2007). Mosaic tetracycline resistance genes are widespread in human and animal fecal samples. *Antimicrob. Agents Chemother*. 51 (3), 1115–1118. doi: 10.1128/AAC.00725-06.

Strommenger, B., Kettlitz, C., Werner, G., Witte, W. (2003). Multiplex PCR assay for simultaneous detection of nine clinically relevant antibiotic resistance genes in *Staphylococcus aureus*. *J. Clin. Microbiol.* 41 (9), 4089–4094. doi: 10.1128/JCM.41.9.4089-4094.2003.

Vakulenko, S.B., Donabedian, S.M., Voskresenskiy, A.M., Zervos, M.J., Lerner, S.A., Chow, J.W. (2003). Multiplex PCR for detection of aminoglycoside resistance genes in enterococci. *Antimicrob. Agents Chemother.* 47 (4), 1423–1426. doi: 10.1128/AAC.47.4.1423-1426.2003.

Wang, Y., Lv, Y., Cai, J., Schwarz, S., Cui, L., Hu, Z., et al. (2015). A novel gene, *optrA*, that confers transferable resistance to oxazolidinones and phenicols and its presence in *Enterococcus faecalis* and *Enterococcus faecium* of human and animal origin. *J. Antimicrob. Chemother.* 70 (8), 2182–2190. doi: 10.1093/jac/dkv116.
