# Supplementary figures and images for: Invasive Streptococcus suis isolated in Spain contain a highly promiscuous and dynamic resistome
Source: Front Cell Infect Microbiol. 2024 Jan 22;13:1329632. doi: 10.3389/fcimb.2023.1329632 (PMC10839070; doi:10.3389/fcimb.2023.1329632)

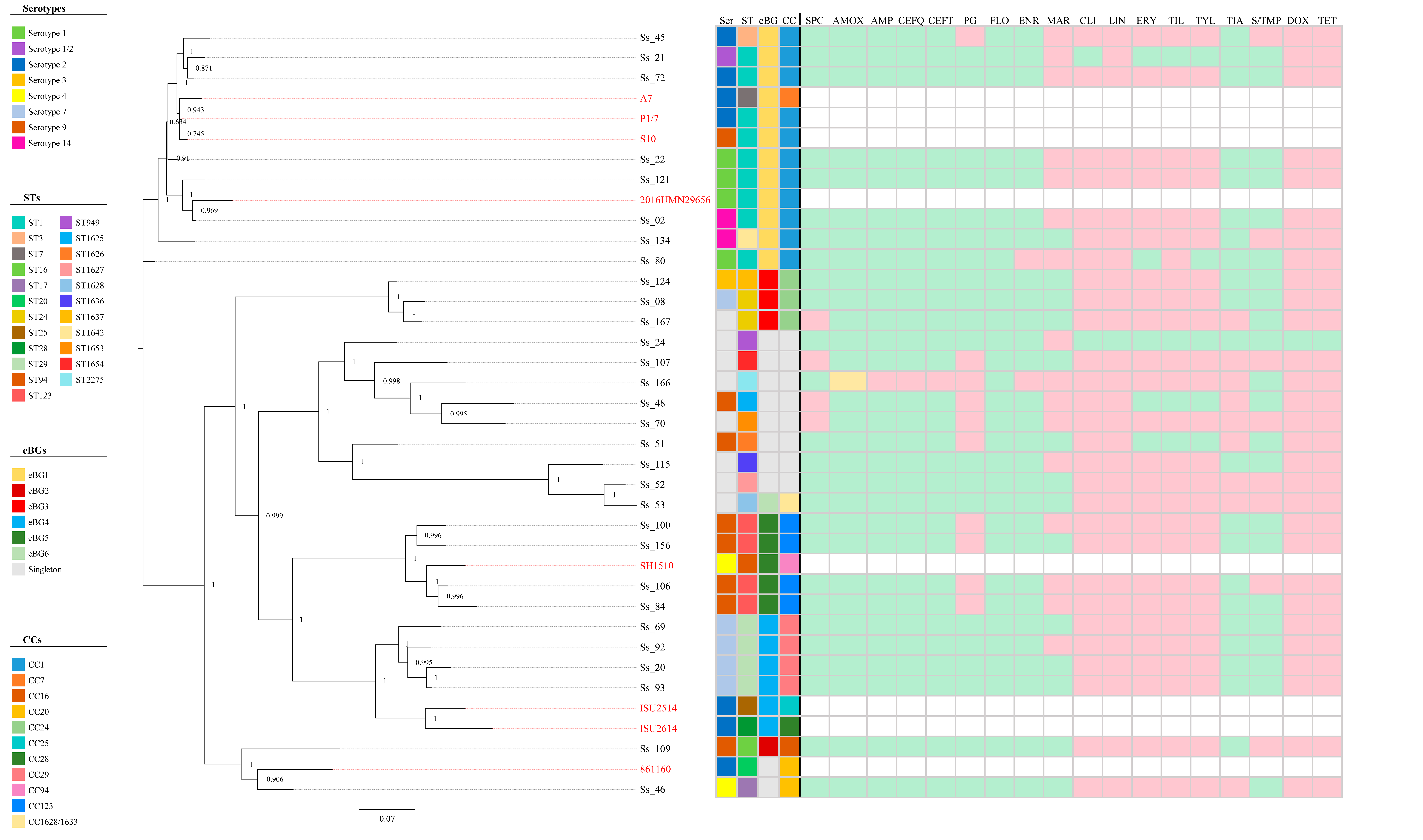

Supplement: Supplementary Figure 1 — Maximum-likelihood phylogenetic tree constructed with the nucleotide sequences of the 30 genomes of our S. suis collection (colored in black) and 8 reference genomes (colored in red). The genetic characteristics of serotype, ST, eBG and CC are given in the first 4 columns (color code as indicated in the figure legend). From column 5 onwards, the resistance profile to the 17 antimicrobials tested is shown in green when the isolate is susceptible, red when is resistant, and yellow when it has intermedia resistance. ST, Sequence Type; eBG, eBurst Group; CC, Clonal Complex; ser, Serotype; SPE, Spectinomycin; AMOX, Amoxicillin; AMP, Ampicillin; CEFQ, Cefquinome; CEFT, Ceftiofur; PG, Penicillin G; FLO, Florfenicol; ENR, Enrofloxacin; MAR, Marbofloxacin; CLI, Clindamycin; LIN, Lincomycin; ERY, Erythromycin; TIL, Tilmicosin; TYL, Tylosin; TIA, Tiamulin; S/TMP, Sulfamethoxazole/Trimethoprim; DOX, Doxycycline; TET, Tetracycline. [file Image_1.jpeg]

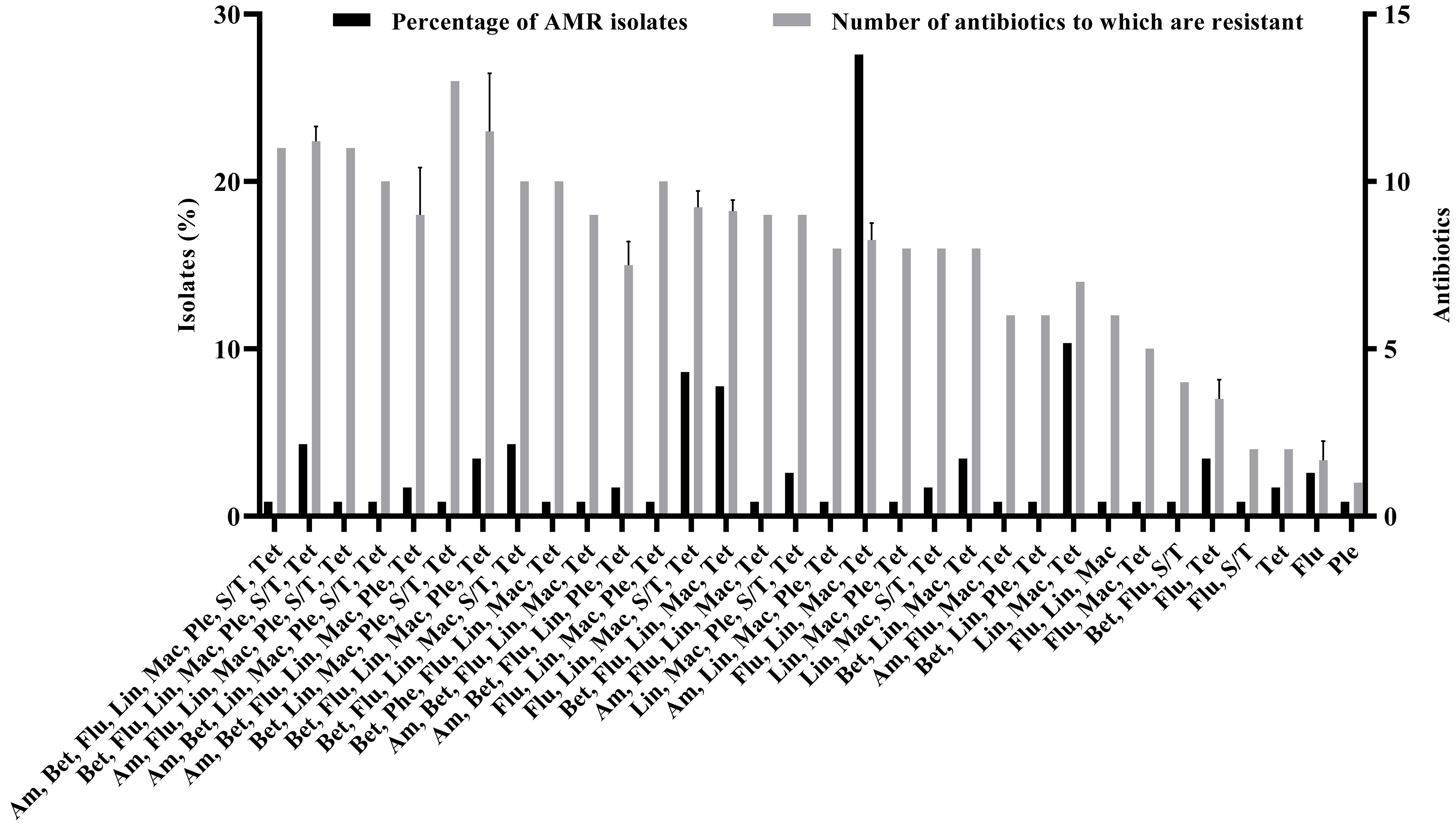

Supplement: Supplementary Figure 2 — Distribution of multi-resistance patterns identified in 116 invasive S. suis isolates. The number of antibiotic families and the percentage of isolates in each pattern is indicated. [file Image_2.jpeg]

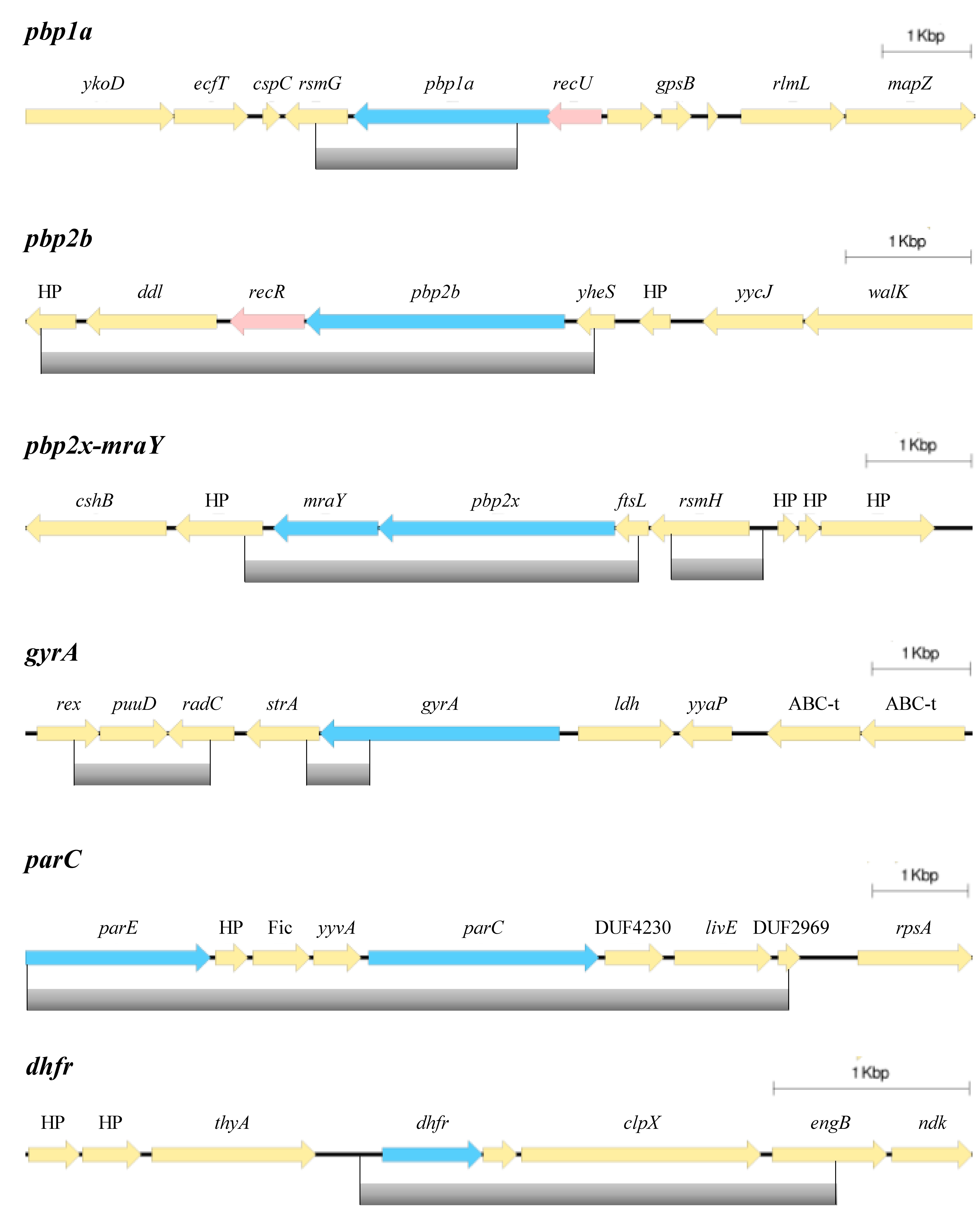

Supplement: Supplementary Figure 4 — Genetic organization of different genes involved in AMR. The shadow indicates sequences where recombination breakpoints were estimated. HP, Hypothetical protein; ABC-t, ABC transporter ATP-binding protein. [file Image_4.jpeg]
